# ﻿Bringing order to a complex system: phenotypic and genotypic evidence contribute to the taxonomy of *Tityus* (Scorpiones, Buthidae) and support the description of a new species

**DOI:** 10.3897/zookeys.1075.67459

**Published:** 2021-12-07

**Authors:** Jairo A. Moreno-González1, Ricardo Pinto-da-Rocha1, Jonas E. Gallão2

**Affiliations:** 1 Instituto de Biociências – Universidade de São Paulo, Departamento de Zoologia. Rua do Matão, travessa 14, 321, 005508-900, São Paulo, Brazil Universidade de São Paulo São Paulo Brazil; 2 Laboratório de Estudos Subterrâneos, Departamento de Ecologia e Biologia Evolutiva, Universidade Federal de São Carlos, Rodovia Washington Luís, km 235, 13565-905 São Carlos, Brazil Universidade Federal de São Carlos São Carlos Brazil

**Keywords:** Cave, Neotropics, scorpion, South America, state of Goiás

## Abstract

We present a molecular phylogenetic analysis including a survey for overlooked phenotypic characters. Based on both analysis and characters a new cave-dwelling species is described: Tityus (Tityus) spelaeus**sp. nov.** from the Russão II cave, Posse, state of Goiás, Central Brazil. Characters such as the glandular regions of the female pectinal basal piece and basal middle lamellae of pectines, and the distribution of the ventral setae of telotarsi I–IV proved to be useful to constructing the taxonomy of species and species groups of *Tityus*. The new species is a member of the *Tityustrivittatus* species-group of Tityus (Tityus) and can be readily recognized by the immaculate coloration pattern and the more developed glandular region on the female pectinal basal piece. In addition, we provide a discussion of the phylogenetic relationships observed within *Tityus*, on the relevance of the new phenotypic characters to the modern taxonomy of the genus *Tityus*, and to the records of Brazilian cave scorpions.

## ﻿Introduction

Among the Neotropical buthid genera, *Tityus* C. L. Koch, 1836 represents the most diverse genus, with more than 220 species (Francke and Stockwell 1987; Fet and Lowe 2000; Lourenço 2006; Souza et al. 2009; Lourenço 2015). The distribution of the genus is broad, ranging from Dominican Republic to Central Argentina (Francke and Stockwell 1987; Armas and Antún 2004; Souza et al. 2009). *Tityus* contains several species that have been considered dangerous to humans due to their potent venoms and involvement in scorpionism (Lourenço 2011, 2015; Borges and Graham 2016). Nevertheless, despite their richness, wide distribution, and medical importance, a detailed phenotypic study of *Tityus* species is lacking, rendering the genus as one of the most taxonomically problematic in the order (Junior 1932; Fet and Lowe 2000; Souza et al. 2009; Ojanguren-Affilastro et al. 2017b; Moreno-González et al. 2019; Ojanguren-Affilastro et al. 2021).

Great challenges need to be overcome before the taxonomy of *Tityus* can be fully resolved. Currently, the genus is one of the most difficult groups to work with, in view of the large number of species that are phenotypically similar, and the ineffectiveness of the somatic characters used to delimit species-groups. For example, Lourenço (2006) proposed the following five subgenera based on coloration; total size; degree of dilation of the basal middle lamellae of the female pectines; shape of the subaculear tubercle, and development of the fulcra of the pectines: Tityus (Archaeotityus) Lourenço, 2006, Tityus (Atreus) Gervais, 1843, Tityus (Brazilotityus) Lourenço, 2006, Tityus (Caribetityus) Armas & Marcano Fondeur, 1992, and Tityus (Tityus) Koch, 1836. Ever since, the monophyly of these groups has not been rigorously tested in a phylogenetic framework, and informal taxonomic categories, such as species-groups, are still prevalent (e.g., Borges et al. 2010; Ojanguren-Affilastro et al. 2017b).

Species-level distinctions are also problematic in *Tityus*. For the most part, species are defined based on the following characters: **i)** total body size; **ii)** coloration pattern; **iii)** pectinal tooth number; **iv)** number of oblique rows in the movable finger of pedipalp chela; **v)** morphometric ratios (particularly in males); **vi)** development and array of carinae of metasoma and pedipalp, and **vii)** shape of the subaculear tubercle (e.g., Lourenço 1984, 2002a, 2002b). However, some of these somatic characters, especially the meristic and morphometric characters, frequently overlap among different species, which makes it difficult to set species boundaries (e.g., Prendini 2001; Teruel and García 2008a, 2008b; Moreno-González et al. 2019).

The problems mentioned above, added to the fact that some species were described based on juveniles (e.g., *T.adisi* Lourenço & Pézier, 2002; *T.canopensis* Lourenço & Pézier, 2002), are worsened by the fact that there are few taxonomic publications including thorough phenotypic descriptions that incorporate genotypic data, comparative diagnoses, and imaging of different character states (e.g., pictures under UV light).

There have been a few modern taxonomic revisions of *Tityus*. For example, recently, Moreno-González et al. (2019) tested traditional (e.g., pectinal tooth and movable finger denticle row counts; morphometric ratios) and new (e.g., glandular region in the pectinal basal piece of females and metasomal macrosetae) phenotypic characters to distinguish among Colombian species of Tityus (Archaeotityus). On the other hand, few phylogenies have included a small number of terminals of *Tityus* (e.g., Borges et al. 2010; Borges and Graham 2016; Ojanguren-Affilastro et al. 2017a; Ojanguren-Affilastro et al. 2021) and several terminals only once (e.g., Román et al. 2018). These studies have either used Sanger sequences only or analyzed morphological characters together with Sanger sequences (e.g., Esposito et al. 2017, 2018). Ojanguren-Affilastro et al. (2017b) used integrative taxonomy and different sources of evidence, such as Sanger sequences, phenotypic characters, and karyotypes, to support the description of a new species: *Tityuscurupi* Ojanguren-Affilastro, Adilardi, Cajade, Ramõarez, Ceccarelli & Mola, 2017 from Northeastern Argentina. More recently, Ojanguren-Affilastro et al. (2021) used phenotypic characters and a molecular phylogeny, based on Sanger sequences, to redescribe *Tityustrivittatus* Kraepelin, 1898 and to describe a new species from Argentina: *Tityuscarrilloi* Ojanguren-Affilastro, 2021. However, these efforts are far from sufficient and more contributions incorporating phenotypic and genotypic evidence are urgently needed to improve our current knowledge of *Tityus*.

### ﻿Cave scorpions

How to classify subterranean organisms based on their restriction/adaptation to the cave habitat has been a matter of debate for a long time (see Trajano and Carvalho 2017 for a review of the most used classification of subterranean organisms). The most popular classification follows Schiner (1854), as emended by Racovitza (1907). It encompasses three categories: trogloxenes, troglophiles, and troglobites. More recently, Trajano (2012) added metapopulation concepts to the Schiner-Racovitza system as follows: a trogloxene source is a population in epigean habitats using subterranean resources; a troglophile source population occurs both in epigean and hypogean habitats, and there is gene flow between habitats; a troglobite source population inhabits exclusively subterranean habitats.

Arachnids (except Solifugae and Thelyphonida) are common in subterranean environments. Cave-dwelling taxa can be found among Acari, Amblypygi, Araneae, Opiliones, Palpigradi, Pseudoscorpiones and, to lesser extent, Ricinulei, Schizomida and Scorpiones (Trajano 1987; Pinto-da-Rocha 1995; Reddell 2012). Few species of scorpions inhabit subterranean habitats compared to other groups mentioned above, but all those scorpions are top predators (Volschenk and Prendini 2008; Reddell 2012).

Troglobitic scorpions are globally rare (Volschenk and Prendini 2008; Sissom and Reddell 2009; Lourenço and Duhem 2010; Reddell 2012; Lourenço and Pham 2013; Gallão and Bichuette 2016). Volschenk and Prendini (2008) redefined the concept of a troglobitic scorpion to species that are restricted to caves and exhibit remarkable troglomorphisms. The following are commonly recognized troglomorphic scorpion features: **i)** reduction or absence of ocelli (median and/or lateral); **ii)** absence of pedal spurs (prolateral and retrolateral); **iii)** reduction of pigmentation and sclerotization; and **iv)** attenuation of legs, pedipalps, and telson vesicle (Volschenk and Prendini 2008). Under this definition, a large proportion of the scorpion species previously recorded to be cave inhabitants fall into the trogloxene or troglophile categories (Lourenço 1981; Lourenço and Francke 1985; Volschenk and Prendini 2008).

Buthidae, the largest scorpion family (~1263 species) (Rein 2021), has few records from subterranean habitats (Volschenk and Prendini 2008; Gallão and Bichuette 2016; Prendini et al. 2021), whereas the buthid genus *Tityus*, the most diverse scorpion genus, has only nine species recorded from caves: Tityus (Tityus) blaseri Mello-Leitão 1931 (Brazil) [probably troglophile]; Tityus (Tityus) confluens bodoquena Lourenço, Cabral & Ramos, 2004 (Brazil) [troglophile]; Tityus (Tityus) demangeiLourenço 1981 (Ecuador) [probably trogloxene]; Tityus (Tityus) jussarae Lourenço, 1988 (Ecuador) [trogloxene]; Tityus (Tityus) grottoedensis Botero-Trujillo & Flórez, 2014 (Colombia) [probably troglophile]; Tityus (Atreus) magnimanus Pocock 1897 (Venezuela) [troglophile or trogloxene]; Tityus (Tityus) monaguensisGonzález-Sponga 1974 (Venezuela) [troglophile or trogloxene]; Tityus (Atreus) obscurus Gervais 1843 (Brazil) [probably accidental], and Tityus (Tityus) stigmurus (Thorell 1876) (Brazil) [majority of records probably accidental, but with troglophile populations in caves of the state of Sergipe (M.E. Bichuette pers. comm.)] (González-Sponga 1974; Lourenço 1981; Trajano 1987; Trajano and Moreira 1991; Pinto-da-Rocha 1995; Lourenço et al. 1997, 2004; Volschenk and Prendini 2008; Lourenço and Duhem 2010; Botero-Trujillo and Flórez 2014). Recently, Prendini et al. (2021) classified *T.grottoedensis* as trogloxene, and *T.demangei*, *T.magnimanus* and *T.monaguensis* as accidental. However, there are scarce field observations on the dependence on and use of subterranean habitats by most *Tityus* species.

In this contribution, we present a phylogenetic hypothesis including a survey for overlooked phenotypical characters. Based on both analysis and characters a new cave-dwelling species is described: *Tityusspelaeus* sp. nov. from Russão II cave, Posse, state of Goiás, Central Brazil. Also, we discuss the phylogenetic relationships observed within *Tityus*, on the relevance of the new phenotypic characters in the modern taxonomy of the genus, and to the records of Brazilian cave scorpions.

## ﻿Materials and methods

### ﻿Materials

The type-material of the new species is housed in the Laboratório de Estudos Subterrâneos (**LES/UFSCar**), São Carlos, Brazil (Curator: Dr. Maria E. Bichuette), in the Museu de Zoologia da Universidade de São Paulo (**MZSP**), São Paulo, Brazil (curator: Dr. Ricardo Pinto-da-Rocha), and the Cryo Collection of the Laboratory of Evolution and Systematics of Arachnids (**IBALCC-RPDR**), Instituto de Biociências, Universidade de São Paulo, São Paulo, SP, Brazil (**IB-USP**). Other materials are listed in Appendix 1.

According to Lourenço (2019) the type material of *Tityusacutidens* Mello-Leitão, 1933 (MNRJ 27781); *Tityusblaseri* Mello-Leitão, 1931 (MNRJ 11282); *Tityusthelyacanthus* Mello-Leitão, 1933 (MNRJ 11280); *Tityusuniformis* Mello-Leitão, 1931 (MNRJ 7041), and *Tityusjeanvellardi* Lourenço, 2001 (MNRJ 7135) were destroyed during the fire that in 2018 consumed the Museu Nacional/ Universidade Federal do Rio de Janeiro (MNRJ). However, about half of the type materials of *Tityus* had been requested on loan, by the first and second authors in 2016 and survived the fire. This loan included all the aforementioned species except for *T.uniformis*, in addition to the following species: *Tityusaba* Candido, Lucas, de Souza, Diaz & Lira-da-Silva, 2005 (MNRJ 7655); *Tityuscarvalhoi* Mello-Leitão, 1945 (MNRJ 7043); *Tityusdasyurusfulvipes* Mello-Leitão, 1945 (MNRJ 7051); *Tityusevandroi* Mello-Leitão, 1945 (MNRJ 7049); *Tityusintermediusiophorus* Mello-Leitão, 1931 [= *Tityusthelyacanthus*] (MNRJ 11280); *Tityuskuryi* Lourenço, 1997 (MNRJ 7035); *Tityusmaranhensis* Lourenço, de Jesus Junior & Limeira-de-Oliveira, 2006 (MNRJ 11212); *Tityusmartinpaechi* Lourenço, 2001 (MNRJ 7077); *Tityusmunozi* Lourenço, 1997 (MNRJ 7036, 7136), and *Tityusnematochirus* Mello-Leitão, 1941 (MNRJ 7052). Other types of *Tityus* species not mentioned here and belonging to the MNRJ were destroyed during the fire.

### ﻿Morphology

Specimens were studied under a Leica MZ75 stereomicroscope with an ocular micrometer. Z-stack pictures under white light and UV light were taken using a Leica MC 170 HD camera. Habitus pictures were taken under white light using a Nikon D3300 digital camera and a 65 mm lens. For Scanning Electron Microscopy (SEM) imaging, a pectine was dissected and cleaned in distilled water with neutral detergent by ultrasound for one minute. After cleaning, the pectine was washed with distilled water and dehydrated via an ethanol concentration gradient (70%, 80%, 90%, 96%, and 100%), giving it 5–15 min in each concentration. Dehydration was completed under critical point drying with the pectine mounted onto a SEM stub using copper tape, after which it was sputter-coated with gold. Stubs were photographed using a Zeiss DSM 940 at Imaging Laboratory of the Instituto de Biociências, Universidade de São Paulo, SP, Brazil (IB-USP). General parameters of pictures were edited with GIMP 2.10 (http://www.gimp.org/), whereas the plates were made with INKSCAPE 1.1 (http://www.inkscape.org/).

General terminology follows Stahnke (1970) and Sissom et al. (1990), except for metasoma and pedipalp carination (Prendini 2000, 2003a), cheliceral dentition in Buthidae (Vachon 1963), trichobothrial notations (Vachon 1974, 1975), nomenclature of the lateral eyes (Loria and Prendini 2014), sternum shape (Soleglad and Fet 2003), and notation of the ventrosubmedian macrosetal count on the leg telotarsi (Francke 1977). Classification for subterranean species follows Trajano (2012).

### ﻿Abbreviations

Pedipalp carinae:

**D** digital;

**DE** dorsoexternal;

**DI** dorsointernal;

**DM** dorsomedian;

**DMA** dorsomarginal;

**DS** dorsal secondary;

**IM** internomedian;

**EM** externomedian;

**ES** external secondary;

**VE** ventroexternal;

**VI** ventrointernal;

**SA** secondary accessory.

Mesosoma, metasoma, and telson carinae:

**DL** dorsolateral;

**DSM** dorsosubmedian;

**ML** median lateral;

**VL** ventrolateral;

**VM** ventromedian;

**VSM** ventrosubmedian.

Others:

**L** length;

**H** height;

**W** width.

### ﻿Taxon sampling

The ingroup taxa comprised 31 terminals of 20 described species of *Tityus* (Table 2). Sequences for 16 terminals were generated for the first time for this study, whereas sequences for 15 other terminals were retrieved from Genbank (Table 2). The type species of three out of five *Tityus* subgenera were included in the analysis: Tityus (Archaeotityus) (i.e., *Tityusclathratus*); Tityus (Atreus) (i.e., *Tityusforcipula*), and Tityus (Tityus) (i.e., *Tityusbahiensis*). The taxon sampling was based on the unpublished results of the first author’s Ph.D. dissertation (Moreno-González 2021) and intend to test the phylogenetic placement of *Tityusspelaeus* sp. nov. The tree was rooted using *Isometrusmaculatus* (DeGeer 1778) following Esposito et al. (2017, 2018).

### ﻿Collection of genotypic characters

We extracted genomic DNA from leg tissues using the protocol of Fetzner (1999) and kept voucher specimens in the IBALCC-RPDR. Extractions were quantified using a Thermo Scientific Nanodrop spectrophotometer. Genomic DNA was used as a template to amplify four loci (12S rRNA, 16S rRNA, 28S rRNA, and COI) using universal primers (Table 1) and the protocol described by Pinto-da-Rocha et al. (2014): PCR reactions had a volume of 25 μL = 13.95 μL Milli-Q H2O, 5 μL PCR buffer (Fermentas), 2 μL MgCl2, 1 μL dNTPs (80 μM) (Fermentas), 1 μL primer (0.4 μM) of each primer, and 0.05 μL GoTaq DNA polymerase (Fermentas). To amplify 28S, we added 1.25 μL dimethyl sulfoxide (DMSO) to the final solution. We conducted PCR reactions in an Eppendorf Mastercycler gradient thermal cycler with the following set-up (temperature/ time): 95 °C/ 5 min (initial denaturation), followed by 35 cycles of 95 °C/ 30s (denaturation), 30s at different temperatures for each set of primers (annealing) (see Table 1), and 72 °C/ 60s (extension), ending with 72 °C/ 7 min (final extension) and an infinite hold of 4 °C (cooling). For specimens and markers that did not amplify, we used Phusion High-Fidelity DNA Polymerase Taq (Finnzymes), following the manufacturer’s protocol for 1 μL DNA extract. For COI degenerated primers, we used a touch-down PCR with the parameters proposed by Astrin et al. (2016).

**Table 1.** List of primers used to amplify DNA sequences of *Tityus* species. Abbreviations: **F** forward **R** reverse **T** temperature.

Locus	Primer	Sequences	Direction	Annealing (T, °C)	Reference
COI	LCO1490-jj2	5’- CHA CWA AYC AYA ARG AYA TYG G	F	49.3–62.0	Astrin et al. (2016)
COI	HCO2198-jj2	5’- ANA CTT CNG GRT GNC CAA ARA ATC A	R	57.9–66.7	Astrin et al. (2016)
12S	12Sai	5’- AAA CTA GGA TTA GAT ACC CTA TTA T	F	52.3	Kocher et al. (1989)
12S	12Sbi	5’- AAG AGC GAC GGG CGA TGT GT	R	64.6	Kocher et al. (1989)
12S	12Sop2r	5’ CCC TTA AAY YTA CTT TGT TAC GAC C	R	50	Pinto-da-Rocha et al. (2014)
16S	16Sbr	5’- CTC CGG TTT GAA CTC AGA TCA	F	57.7	Simon et al. (1994)
16S	16S_F	5’- CGA TTT GAA CTC AGA TCA	F	49.3	Gantenbein et al. (1999)
16S	16Sbr_mod	5’- GTG CAA AGG TAG CAT AAT CA	R	53.7	Gantenbein et al. (1999)
28S	28Sa (Sad3)	5’- GAC CCG TCT TGA AAC ACG GA	F	60.3	Whiting et al. (1997)
28S	28Srd5b	5’- CCA CAG CGC CAG TTC TGC TTA C	R	64.2	Schwendinger and Giribet (2005)
28S	28SBout	5’- CCC ACA GCG CCA GTT CTG CTT ACC	R	68	Schulmeister (2003)

PCR amplifications were checked using electrophoresis of agarose gel (2% agarose). Positive amplifications were purified using Agencourt Ampure XP (Beckman Coulter), then quantified using a Thermo Scientific NanoDrop spectrophotometer. We prepared sequencing reactions with the BigDye Terminator v3.1 Cycle Sequencing Kit (Applied Biosystems), precipitated PCR products with sodium acetate, and sequenced using an ABI PRISM 3100 Genetic Analyzer/HITACHI (Applied Biosystems). Sequence editing (e.g., primer trimming) and contiguous sequence generation were made on AB1 files using Geneious R11 (http://www.geneious.com). Consensus sequences were checked against the NCBI nucleotides database using the BLAST algorithm to detect for possible contaminations. Sequences without contamination were grouped into FASTA files separated by loci, and then inspected to detect potential reverse complemented sequences.

### ﻿Phylogenetic analysis

#### Sequence alignment

Ribosomal gene and intron sequences (12S rDNA, 16S rDNA, and 28S rDNA) were aligned using the E-INS-i algorithm of MAFFT (Katoh and Standley 2013). Protein-coding gene sequences (Cytochrome *c* Oxidase I- COI) were aligned using the L-INS-i algorithm. The COI alignment was translated and inspected for stop codons using Geneious R11 (http://www.geneious.com). A single concatenated matrix composed of all sequences was created using SequenceMatrix (Vaidya et al. 2011) and exported as a NEXUS file. The final file was visualized and edited in Geneious R11, where leading and trailing gaps were substituted by ‘N’ since they most probably corresponded to differential sequencer reading starting and ending points.

**Table 2.** List of terminals, voucher specimens, and sequences (GenBank accession numbers indicated) used in the phylogenetic analysis of *Tityus*. (*) Sequence already available on GenBank before the outset of this study. Abbreviations: NA, not applicable.

Species	Subgenus	Voucher	12S	16S	28S	COI
*Isometrusmaculatus* (DeGeer, 1778)	NA	AMNH LP 1798	KY981825.1*	KY981921.1*	KY982111.1*	KY982207.1*
*Tityusargentinus* Borelli, 1899	*Tityus*	MACN Ar 35705	NA	KY674452*	KY674474*	KY674493*
*Tityusbahiensis* (Perty, 1833)	*Tityus*	IBALCC RPDR 00281	OK493267	OK493246	OK493233	OK561906
*Tityusblaseri* Mello-Leitão, 1931	*Tityus*	IBALCC RPDR 00027	OK493254	OK493248	OK493221	OK561901
*Tityusblaseri* Mello-Leitão, 1931	*Tityus*	IBALCC RPDR 00114	OK493256	OK493238	OK493223	OK561904
*Tityusbrazilae* Lourenço & Eickstedt, 1984	*Tityus*	IBALCC RPDR 00168	OK493258	OK493239	OK493225	OK561902
*Tityusbrazilae* Lourenço & Eickstedt, 1984	*Tityus*	IBALCC RPDR 00169	OK493259	OK493250	OK493226	OK561894
*Tityusbrazilae* Lourenço & Eickstedt, 1984	*Tityus*	IBALCC RPDR 00199	OK493262	OK493242	OK493228	OK561907
*Tityuscarrilloi* Ojanguren-Affilastro, 2021	*Tityus*	MACN Ar 35713	NA	KY674461*	KY674483*	KY674501*
*Tityuscarvalhoi* Mello-Leitão, 1945	*Tityus*	MACN Ar 35708	NA	KY674455*	KY674477*	KY674495*
*Tityuscharreyroni* Vellard, 1932	*Tityus*	IBALCC RPDR 00112	OK493255	OK493237	OK493222	OK561903
*Tityusclathratus* C. L. Koch, 1844	*Archaeotityus*	IBALCC RPDR 00192	OK493261	OK493241	NA	OK561895
*Tityusconfluens* Borelli, 1899	*Tityus*	MACN Ar 35709	NA	KY674456*	KY674478*	KY674496*
*Tityuscurupi* Ojanguren-Affilastro et al. 2017	*Tityus*	MACN Ar 35693	NA	KY674422*	KY674430*	KY674438*
*Tityuscurupi* Ojanguren-Affilastro et al. 2017	*Tityus*	MACN Ar 35694	NA	KY674423*	KY674431*	KY674439*
*Tityuscurupi* Ojanguren-Affilastro et al. 2017	*Tityus*	MACN Ar 35695	NA	KY674424*	KY674432*	KY674440*
*Tityuscurupi* Ojanguren-Affilastro et al. 2017	*Tityus*	MACN Ar 35723	NA	KY674421*	KY674429*	KY674437*
*Tityuscurupi* Ojanguren-Affilastro et al. 2017	*Tityus*	MACN Ar 35724	NA	KY674457*	KY674479*	KY674497*
*Tityusforcipula* (Gervais, 1843)	*Atreus*	IBALCC RPDR 00256	OK493264	OK493251	OK493230	OK561898
*Tityusobscurus* (Gervais, 1843)	*Atreus*	IBALCC RPDR 00236	OK493263	OK493243	OK493229	OK561905
*Tityuspanguana* Kovařík et al. 2015	*Tityus*	IBALCC RPDR 00268	OK493265	OK493244	OK493231	OK561908
*Tityuspotameis* Lourenço & Giupponi, 2004	*Tityus*	IBALCC RPDR 00275	OK493266	OK493245	OK493232	OK561899
*Tityussastrei* Lourenço & Flórez, 1990	*Atreus*	IBALCC RPDR 00382	OK493268	OK493252	OK493234	OK561897
*Tityusserrulatus* Lutz & Mello, 1922	*Tityus*	IBALCC RPDR 00016	OK493253	OK493247	OK493220	OK561900
*Tityussoratensis* Kraepelin, 1912	*Tityus*	MACN Ar 35712	NA	KY674460*	KY674482*	KY674500*
*Tityusspelaeus* sp. nov.	*Tityus*	IBALCC RPDR 00116	OK493257	OK493249	OK493224	NA
*Tityusstigmurus* (Thorell, 1876)	*Tityus*	IBALCC RPDR 00170	OK493260	OK493240	OK493227	OK561896
*Tityusuruguayensis* Borelli, 1901	*Tityus*	MACN Ar 35714	NA	KY674425*	KY674433*	KY674442*
*Tityusuruguayensis* Borelli, 1901	*Tityus*	MACN Ar 35715	NA	KY674462*	KY674484*	KY674502*
*Tityusuruguayensis* Borelli, 1901	*Tityus*	MACN Ar 35716	NA	KY674426*	KY674434*	KY674443*
*Tityusuruguayensis* Borelli, 1901	*Tityus*	MACN Ar 35717	NA	KY674427*	KY674435*	KY674444*
*Tityusuruguayensis* Borelli, 1901	*Tityus*	MACN Ar 35718	NA	KY674428*	KY674436*	KY674445*

#### Tree search

Tree search was conducted in IQTREE using the maximum likelihood (ML) criterion (Minh et al. 2020), with the command line: “*iqtree -s matrix.nex -st DNA -spp partitions.nex -pre matrix.nex -m MFP -bb 1000 -ninit 1000* -*nt 3*”. Molecular evolution models were selected for each partition based on the BIC value criterion. Ultrafast Bootstrap values were calculated in IQTREE after 1000 replications. Tree files were edited with Figtree v1.4.4 (https://github.com/rambaut/figtree/) and INKSCAPE 1.1 (http://www.inkscape.org/).

## ﻿Results

### ﻿Phylogenetic relationships

**Figure 1.**
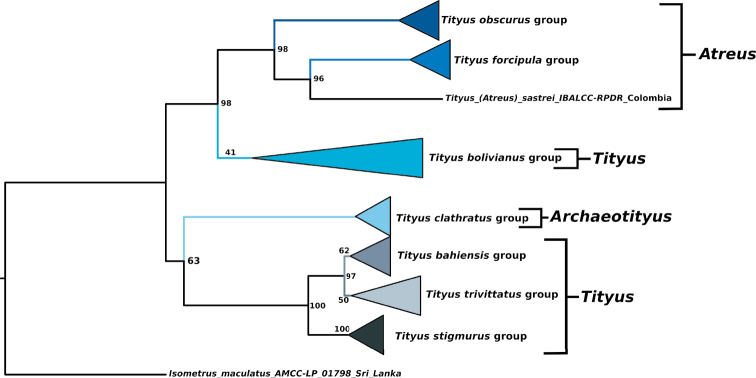
Phylogeny of *Tityus* representatives from South America obtained by analysis of DNA sequences (12S rDNA, 16S rDNA, 28S rDNA, and Cytochrome *c* Oxidase I- COI). Maximum likelihood tree (Log-likelihood= -12896.086), showing species-groups and subgenera. Values on nodes correspond to ultrafast-bootstrap (Ubst) values.

**Figure 2.**
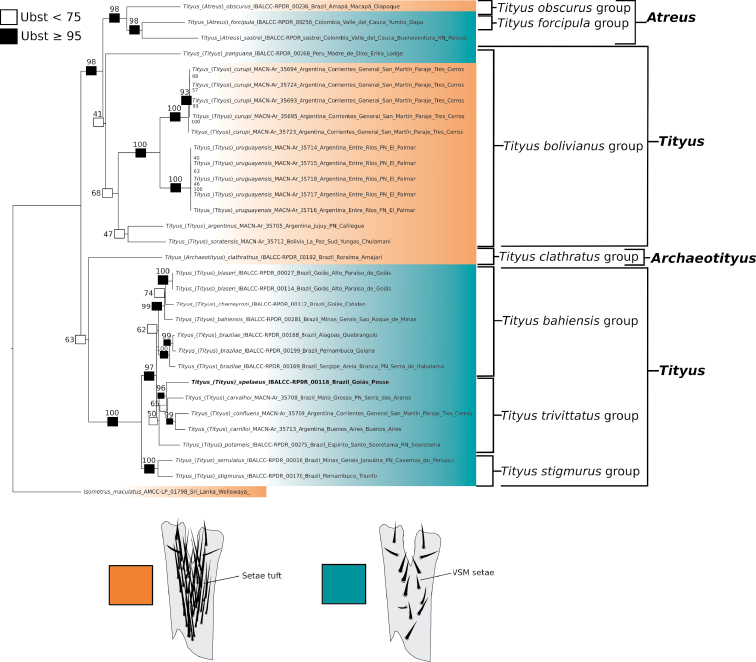
Phylogeny of *Tityus* representatives from South America obtained by analysis of DNA sequences (12S rDNA, 16S rDNA, 28S rDNA, and Cytochrome *c* Oxidase I- COI), showing the distribution of the characters states of the ventral setae of telotarsi I–IV (orange: an irregularly distributed tuft of setae (type I); turquoise: two ventro-submedian rows of setae (type II)) across different *Tityus* subgenera and species-groups. Boxes on branches and associated values correspond to ultrafast-bootstrap (Ubst) values. Observations= Tityus (Tityus) spelaeus sp. nov. is marked in bold.

**Figure 3.**
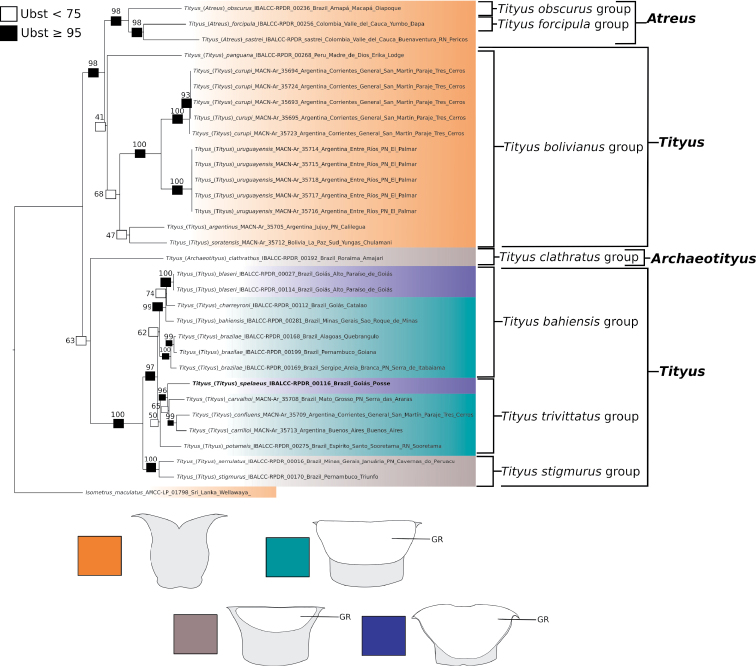
Phylogeny of *Tityus* representatives from South America obtained by analysis of DNA sequences (12S rDNA, 16S rDNA, 28S rDNA, Cytochrome *c* Oxidase I), showing distribution of the characters states exhibited by the female basal pectinal piece (orange: GR absent; grey: medium-sized GR; turquoise: relatively large GR; blue: very large GR) across different *Tityus* subgenera and species-groups. Boxes on branches and associated values correspond to ultrafast-bootstrap (Ubst) values. Observations= Tityus (Tityus) spelaeus sp. nov. is marked in bold. Abbreviations= GR, glandular region.

The tree log-likelihood score was -12896.086. The best-fit models per molecular partition were TIM2+F+G4 (12S), TIM2+F+I+G4 (16S), TNe+R2 (28S), and TIM+F+I+G4 (COI). Based on the phylogenetic hypothesis that was obtained (Figs 1–3), the subgenus Tityus (Tityus), as currently defined, is polyphyletic and composed of at least three main lineages: one lineage includes the species-groups *T.bahiensis* (ultrafast bootstrap value (Ubst)= 74), *T.stigmurus* (Ubst= 100), and *T.trivittatus* (Ubst= 50), a second lineage corresponds to the *T.bolivianus* species-group (Ubst= 41), and a third lineage is that of the species *T.sastrei* (Figs 1–3). In order to arrive at a monophyletic Tityus (Tityus), it will be necessary to remove the *T.bolivianus* species-group from this subgenus and transfer *T.sastrei* to Tityus (Atreus) (Figs 1–3). We transferred *T.sastrei* to Tityus (Atreus), but think that additional data are needed to propose an appropriate subgeneric designation of the *T.bolivianus* species-group.

Tityus (Archaeotityus) was recovered as the sister group (Ubst= 63) of a clade containing three Tityus (Tityus) species-groups (*T.bahiensis*, *T.stigmurus*, and *T.trivittatus* species-groups). On the other hand, a new species here described, Tityus (Tityus) spelaeus sp. nov., was recovered as a member of the *T.trivittatus* species-group (Ubst= 87) and is closely related to *T.carrilloi*, *T.carvalhoi*, and *T.confluens* (Figs 2, 3). Similarly, Tityus (Atreus) was recovered as polyphyletic with one clade composed of T. (Atreus) forcipula, T. (Atreus) sastrei, and T. (Atreus) obscurus (Ubst= 98), and another clade composed of T. (Atreus) brazilae (Ubst= 100) which is nested inside the *T.bahiensis* species-group (Figs 1–3). Therefore, to make Tityus (Atreus) a monophyletic group, *T.brazilae* is here formally transferred to the *T.bahiensis* species-group of Tityus (Tityus) (Figs 2, 3). Finally, the *T.bolivianus* species-group appeared as the sister group (Ubst= 98) of the clade composed of Tityus (Atreus) (Figs 2, 3).

### ﻿Phenotypic characters

#### Ventral setae of telotarsi I–IV

**Figure 4.**
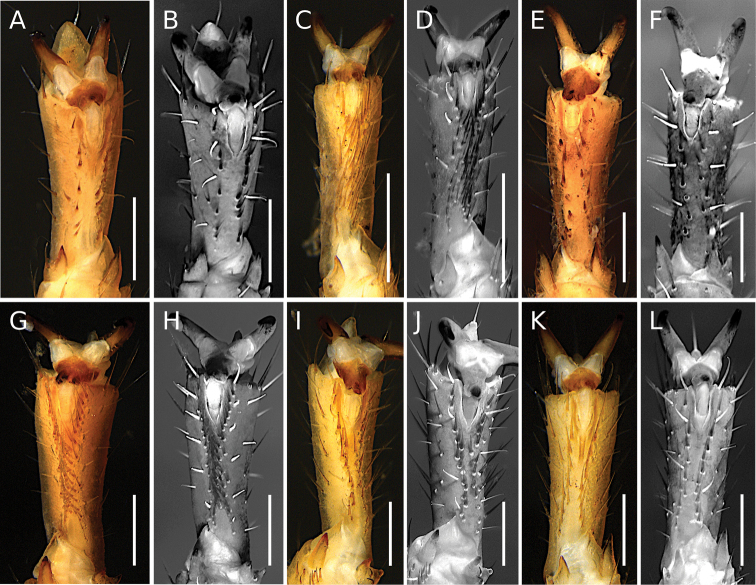
*Tityus* C. L. Koch, 1836, telotarsi IV, showing ventral macrosetae **A, C, E, G, I, K** white light **B, D, F, H, J, L** UV light **A, B**Tityus (Tityus) brazilae Lourenço & Eickstedt, 1984 (type II) (MZSP 75619) **C, D**Tityus (Archaeotityus) clathratus C. L. Koch, 1844 (type I) (MZSP 31468) **E, F**Tityus (Atreus) forcipula (Gervais, 1843) (type II) (MZSP) **G, H**Tityus (Atreus) obscurus Gervais, 1843 (type I) (MNRJ 07610) **I, J**Tityus (Tityus) serrulatus Lutz & Mello, 1922 (type II) (MZSP 28205) **K, L**Tityus (Tityus) spelaeus sp. nov. (MZSP 74633) (type II). Observations = telotarsi I–IV ventral setae distribution: Type I = tuft of irregularly distributed setae. Type II = two discrete ventrosubmedian rows of setae. Scale bars: 500 μm.

We observed that the distribution of the ventral setae of telotarsi I–IV in *Tityus* can exhibit two states: **i)** an irregularly distributed tuft of setae (type I) (Figs 2, 4C, D, G, H) or **ii)** two ventro-submedian rows of setae (type II) (Figs 2, 4A, B, E, F, I–L) (Table 3). According to our phylogenetic hypothesis (Fig. 1) and a comprehensive total evidence analysis (e.g., Moreno-González 2021), the distribution of ventral macrosetae on telotarsi is highly homoplastic (Figs 2, 4; Table 3). For example, species-groups such as *T.bahiensis*, *T.bolivianus* [in part: *T.panguana*], *T.forcipula*, *T.stigmurus*, and *T.trivittatus*, and the species *T.sastrei* share ventral setation type II on telotarsi I–IV (Fig. 2; Table 3). Other species-groups such as *T.bolivianus*, *T.clathratus*, and *T.obscurus* exhibit ventral setation type I on telotarsi I–IV (Fig. 2; Table 3). Morphological variations of this character were not observed within the same species or species-group (except for *T.panguana* in the *T.bolivianus* species-group, which exhibited ventral setation type II). However, both Tityus (Atreus) and Tityus (Tityus) exhibited the two character states (Fig. 2).

**Table 3.** Phenotypic characters useful for the taxonomy of *Tityus*. (***) Species here transferred to the indicated subgenus; ventral macrosetae distribution on telotarsi I–IV: **Type I**= tuft of irregularly distributed macrosetae. **Type II**= two discrete ventrosubmedian rows of macrosetae. Abbreviations: BML, basal middle lamellae; D, dilated; NA, not applicable; ND, not dilated; PBP, pectinal basal piece.

Species	Subgenus	Species Group	Telotarsal setae	Females	
				PBP gland	BML
*Isometrusmaculatus* (DeGeer, 1778)	NA	NA	Type II	Absent	ND
*Tityusargentinus* Borelli, 1899	*Tityus*	*T.bolivianus*	Type I	Absent	D= semicircular
*Tityusbahiensis* (Perty, 1833)	*Tityus*	*T.bahiensis*	Type II	First 2/3 of the anterior region	ND
*Tityusblaseri* Mello-Leitão, 1931	*Tityus*	*T.bahiensis*	Type II	More than first 2/3 of the anterior region	ND
*Tityusbrazilae* Lourenço & Eickstedt, 1984***	*Tityus*	*T.bahiensis*	Type II	First 2/3 of the anterior region	ND
*Tityuscarrilloi* Ojanguren-Affilastro, 2021	*Tityus*	*T.trivittatus*	Type II	First 2/3 of the anterior region	ND
*Tityuscarvalhoi* Mello-Leitão, 1945	*Tityus*	*T.trivittatus*	Type II	First 2/3 of the anterior region	ND
*Tityuscharreyroni* Mello-Leitão, 1933	*Tityus*	*T.bahiensis*	Type II	First 2/3 of the anterior region	ND
*Tityusclathratus* C. L. Koch, 1844	*Archaeotityus*	*T.clathratus*	Type I	First anteromedian third	ND
*Tityusconfluens* Borelli, 1899	*Tityus*	*T.trivittatus*	Type II	First 2/3 of the anterior region	ND
*Tityuscurupi* Ojanguren-Affilastro et al. 2017	*Tityus*	*T.bolivianus*	Type II	Absent	D= suboval
*Tityusforcipula* (Gervais, 1843)	*Atreus*	*T.forcipula*	Type II	Absent	D= suboval
*Tityusobscurus* Gervais, 1843	*Atreus*	*T.obscurus*	Type I	Absent	D= semicircular
*Tityuspanguana* Kovařík et al. 2015	*Tityus*	*T.bolivianus*	Type II	Absent	D= semicircular
*Tityuspotameis* Lourenço & Giupponi, 2004	*Tityus*	*T.trivittatus*	Type II	First 2/3 of the anterior region	ND
*Tityussastrei* Lourenço & Flórez, 1990***	*Atreus*	NA	Type II	Absent	D= semicircular
*Tityusserrulatus* Lutz & Melo, 1922	*Tityus*	*T.stigmurus*	Type II	First anteromedian third	ND
*Tityussoratensis* Kraepelin, 1912	*Tityus*	*T.bolivianus*	?	Absent	D= semicircular
*Tityusspelaeus* sp. nov.	*Tityus*	*T.trivittatus*	Type II	More than first 2/3 of the anterior region	ND
*Tityusstigmurus* (Thorell, 1876)	*Tityus*	*T.stigmurus*	Type II	First anteromedian third	ND
*Tityustrivittatus* Kraepelin, 1898	*Tityus*	*T.trivittatus*	Type II	First 2/3 of the anterior region	ND
*Tityusuruguayensis* Borelli, 1901	*Tityus*	*T.bolivianus*	Type I	Absent	D= semicircular

#### Development of pectinal basal piece and basal middle lamellae of female pectines

**Figure 5.**
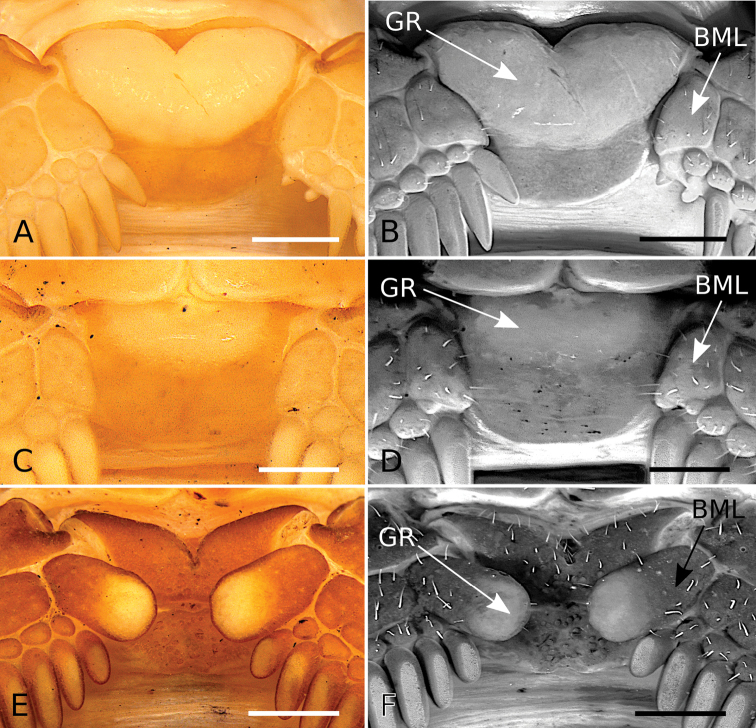
*Tityus* C. L. Koch, 1836, female pectinal basal piece and basal middle lamellae of the pectines, showing glandular regions **A, C, E** White light **B, D, F** UV light **A, B**Tityus (Tityus) brazilae Lourenço & Eickstedt, 1984 (MZSP 75619) **C, D**Tityus (Archaeotityus) clathratus C. L. Koch, 1844 (MZSP 31468) **E, F**Tityus (Atreus) forcipula (Gervais, 1843) (MZSP). Abbreviations: BML, basal middle lamellae; GR, glandular region. Scale bars: 500 μm.

**Figure 6.**
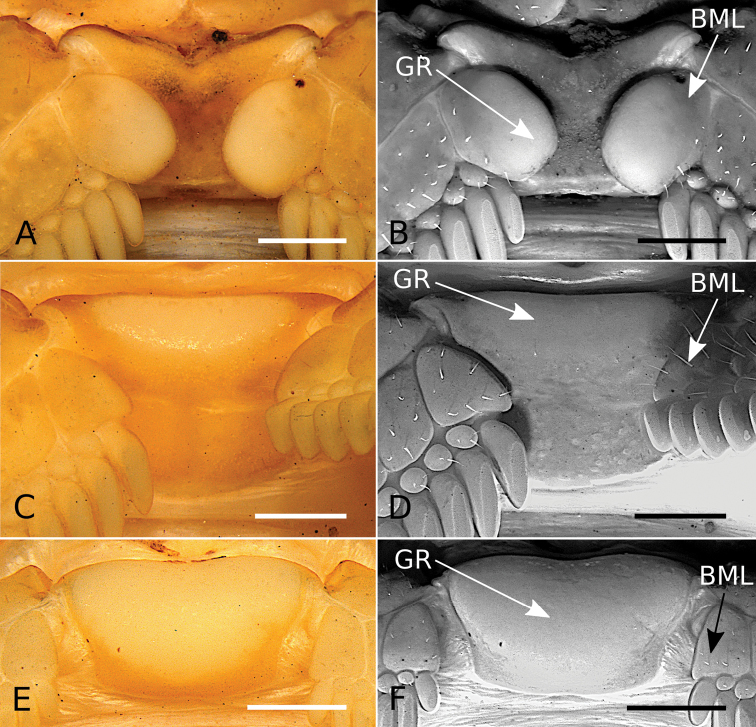
*Tityus* C. L. Koch, 1836, female pectinal basal piece and basal middle lamellae of the pectines, showing glandular regions **A, C, E** White light **B, D, F** UV light **A, B**Tityus (Atreus) obscurus Gervais, 1843 (MNRJ 07610) **C, D**Tityus (Tityus) serrulatus Lutz & Mello, 1922 (MZSP 28205) **E, F**Tityus (Tityus) spelaeus sp. nov. (MZSP 74633). Abbreviations: BML, basal middle lamellae; GR, glandular region of the pectinal basal piece. Scale bars: 500 μm.

The pectinal basal piece of female exhibits the following character states within the examined terminals of *Tityus*: **i)** absence of glandular region (Figs 3, 5E, F, 6A, B; Table 3); **ii)** presence of a relatively large glandular region, occupying a large area of anterior two thirds of the anteromedian region (Figs 3, 5A, B; Table 3); **iii)** presence of a medium-sized glandular region, occupying the anterior third, but absent from the anterolateral margins (Figs 3, 5C, D, 6C, D; Table 3), and **iv)** presence of a very large glandular region, occupying beyond the anterior two thirds of the medial region (Figs 3, 6E, F; Table 3). According to our phylogenetic hypothesis (Fig. 1) and a comprehensive total evidence analysis (e.g., Moreno-González 2021), the character states exhibited by the glandular region of the female pectinal basal piece are highly homoplastic (Figs 3, 5, 6; Table 3).

However, it is noteworthy that in some Tityus (Atreus) (i.e., *Tityusforcipula* see Fig. 5E, F and *T.obscurus* see Fig. 6A, B species-groups and *T.sastrei*) and in the *Tityusbolivianus* species-group, both of which lack a glandular region on the female pectinal basal piece (Fig. 3; Table 3), exhibit dilated middle basal lamellae with glandular regions in the female pectines (Table 3). Conversely, Tityus (Archaeotityus) (i.e., *T.clathratus* species-group) (Figs 3, 5C, D) and Tityus (Tityus) (i.e., *T.bahiensis* (Fig. 5A, B), *T.stigmurus* (Fig. 6C, D), and *T.trivittatus* (Fig. 6E, F) species-groups) present well-developed glandular regions on the female pectinal basal piece (Fig. 3; Table 3), but do not exhibit dilatation of the middle basal lamellae of the female pectines (Table 3). Finally, it is worth mentioning that males of *Tityus* species do not exhibit glandular regions on the pectinal basal piece, with the exception of some species of the *T.androcottoides* species-group (i.e., *T.rebierei*- also females).

### ﻿Taxonomy

#### Family Buthidae C. L. Koch, 1837

##### ﻿Genus Tityus

Taxon classificationAnimaliaScorpionesButhidae

C. L. Koch, 1836

E532E5A9-6476-5A7B-924D-42D7F8C696CE

Tityus C. L. Koch 1836: 33.

##### ﻿Subgenus Tityus (Tityus)

Taxon classificationAnimaliaScorpionesButhidae

C. L. Koch, 1836

800A7F3A-42BB-5B2D-966E-D916A855AADC

Tityus (Tityus) : Lourenço (2006): 57, 58, 60, figures 3–6, 10–13, 22.

###### Type species.

*Scorpiobahiensis* Perty, 1833 by monotypy.

###### Comments.

This subgenus currently includes, among others, all species assigned to the *T.bahiensis* Mello-Leitão, 1945; *T.bolivianus* Kraepelin, 1895; *T.stigmurus* Mello-Leitão, 1945, and *T.trivittatus* Mello-Leitão, 1945 species-groups, according to the classification proposal of Lourenço (2006). In addition to *T.brazilae* Lourenço & Eickstedt, 1984, here transferred to this subgenus (see Discussion). On the other hand, *Tityussastrei* Lourenço & Flórez, 1990 belongs to Tityus (Atreus) and is excluded from Tityus (Tityus) (see Discussion). Finally, according to previous hypotheses and our data, the *T.bolivianus* Kraepelin, 1895 species-group forms an independent clade outside Tityus (Tityus), but additional studies, including the study of the type species of this group, are required to propose a formal taxonomic decision.

##### ﻿ Tityus spelaeus sp. nov.

Taxon classificationAnimaliaScorpionesButhidae

05B3683E-8317-5DC3-9567-AA5195E1784D

http://zoobank.org/3AE5D4E6-C2F1-47A7-9768-046B09B2FF48

Figures 1 , 2 , 3 , 4 , 5 , 6 , 7 , 8 ; Tables 3 , 4 , 5

###### Type material.

Brazil: State of Goiás: ***Holotype*.** Adult female from Posse, Russão II cave, 14°05'05.3"S, 46°23'07.1"W, 01.iv.2007, R. Pinto-da-Rocha leg. (MZSP 74633). ***Paratypes*.** Four adult female paratypes, same data as the holotype (MZSP 74634); eight adult females, same locality as the holotype, 23.iv.2015, J. E. Gallão & C. C. de Paula leg. (LES/UFSCar 14668; LES/UFSCar 14669; LES/UFSCar 14670; LES/UFSCar 14671; LES/UFSCar 14672; LES/UFSCar 14673); four adult females, same locality as the holotype, 01.iv.2007, R. Pinto-da-Rocha et al. (MZSP 52228, 52229, 52230, 52231).

###### Etymology.

The species epithet is a derivative form of the Greek noun, σπήλαιον (Latin: caverna), which means cave, in reference to the subterranean habitat where *Tityusspelaeus* has an established population. It is a noun in apposition.

###### Diagnosis.

(Based on female). This species belongs to the *Tityustrivittatus* species-group (Figs 2, 3). Among members of the group distributed in Brazil (*T.carvalhoi* Mello-Leitão, 1945; *T.charreyroni* Vellard, 1932; *T.confluens* Borelli, 1899; *T.fasciolatus* Pessoa, 1935; *T.jeanvellardi* Lourenço, 2001; *T.karaja* Lourenço, 2016; *T.rupestre* Lourenço, 2019; *T.sylviae* Lourenço, 2005, and *T.trivittatus* Kraepelin, 1898), *Tityusspelaeus* sp. nov. can be readily recognized. *Tityusspelaeus* sp. nov.; *T.carvalhoi*; *T.charreyroni*; *T.confluens*; *T.fasciolatus*; *T.rupestre*, and *T.trivittatus* share a subaculear tubercle small, and acute, pointing towards the tip of the aculeus (Fig. 12A). In contrast, *T.jeanvellardi*; *T.karaja*, and *T.sylviae* exhibit a small and coarse subaculear tubercle that points either towards the tip of the aculeus (*T.sylviae*) or towards the middle of the aculeus (*T.jeanvellardi* and *T.karaja*).

On the other hand, *Tityusspelaeus* sp. nov. and *T.sylviae* share a very large glandular region occupying beyond the anterior two thirds of the medial region of the pectinal basal piece of female pectines (Figs 6E, F, 11). In *T.carvalhoi*; *T.charreyroni*; *T.confluens*; *T.fasciolatus*, and *T.trivittatus* (females of *T.jeanvellardi*; *T.karaja* and *T.rupestre* are unknow) the glandular region occupies a large area of anterior two thirds of the anteriomedian region of the pectinal basal piece of female pectines (e.g., Fig. 5A, B).

**Figure 7.**
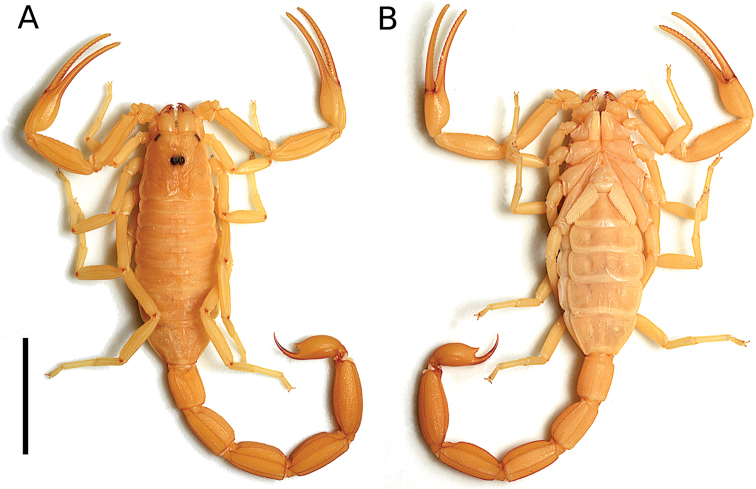
Tityus (Tityus) spelaeus sp. nov., female holotype (MZSP 74633) **A** dorsal view **B** ventral view. Scale bars: 10 mm.

**Figure 8.**
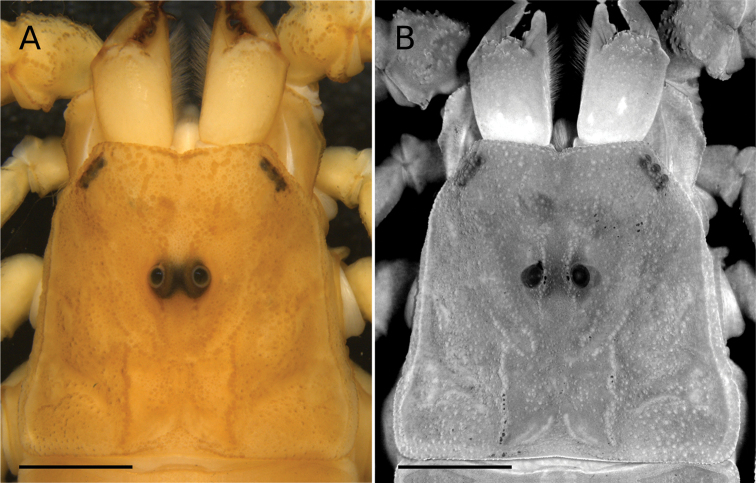
Tityus (Tityus) spelaeus sp. nov., female holotype (MZSP 74633), carapace, dorsal view **A** white light **B** UV light. Scale bars: 2 mm.

Finally, *Tityusspelaeus* sp. nov. and *T.karaja* can be readily distinguished from *T.carvalhoi*; *T.charreyroni*; *T.fasciolatus*; *T.jeanvellardi*;*T.rupestre*; *T.sylviae*, and *T.trivittatus*, based on the presence of residual spots on tergites (Figs 7, 13), and having the carapace (Figs 7, 8A) and chericeral manus immaculate (Fig. 8A). In contrast, *T.charreyroni*; *T.confluens*; *T.fasciolatus*; *T.jeanvellardi*; *T.rupestre*; *T.sylviae*, and *T.trivittatus* have the carapace and tergites moderately covered with brownish spots and the cheliceral manus with reticulations (except *T.jeanvellardi* that exhibit a cheliceral manus immaculate).

###### Remarks.

In an unpublished comprehensive phylogenetic analysis of *Tityus* (Moreno-González 2021), the *Tityustrivittatus* species-group was one of the most morphologically homogeneous species-groups of the genus. In fact, no somatic character of the morphological matrix (~164 chars) was optimized as a synapomorphy in the nodes within the clade representing the *Tityustrivittatus* species-group. Instead, those nodes were solely supported by unambiguous molecular synapomorphies. It is worth mentioning that, although coloration patterns presented high levels of homoplasy, they also showed significant differences at the species level, and the diagnosis of *Tityusspelaeus* sp. nov. is based on this background knowledge. Nonetheless, additional studies including molecular and phenotypical evidence of poorly described species from the Central region of Brazil are required to untangle the phylogeny of this cryptic species complex.

On the other hand, *Tityuskaraja* Lourenço, 2016 was described based on a single male collected in 1929 in the region that corresponds to the northern portion of the state of Goiás, Brazil. According to the brief description of Lourenço (2016), *Tityuskaraja* could share a similar body coloration pattern to that of *T.spelaeus*. However, given that the male of *T.karaja* is almost a hundred years old, the coloration needs to be corroborated with fresh specimens. Despite this, according to Lourenço’s (2016: fig. 5) illustration, the subaculear tubercle of *T.karaja* is conical, small, and coarse, pointing towards the middle of the aculeus, whereas in *T.spelaeus* sp. nov. it points towards the tip of the aculeus (Fig. 12A).

###### Description.

Based on the female holotype (MZSP 74633). Male unknown.

Total length. Female: 53.52 mm (measurements in Table 4).

**Table 4.** Measurements (mm) of *Tityusspelaeus* sp. nov.

Structure	Measure	Female holotype	Female paratype # 1	Female paratype # 2	Female paratype #4	Female paratype #5	Female paratype	Female paratype	Female paratype	Female paratype	Female paratype	Female paratype	Female paratype	Female paratype	Female paratype	Female paratype	Female paratype	Female paratype
		MZSP 74633	MZSP 74633	MZSP 74633	MZSP 74633	MZSP 74633	MZSP 52228	MZSP 52229	MZSP 52230	MZSP 52231	LES014668	LES014669	LES014670	LES014671	LES014672	LES014673	LES014673	LES014673
Total length	–	53.52	51.29	57.89	51.06	51.70	48.30	49.64	51.41	49.90	57.98	53.69	53.84	54.19	54.40	50.75	52.02	54.29
Carapace	length	6.00	5.84	6.40	5.68	5.68	5.57	5.57	5.71	5.57	6.45	5.83	5.98	6.06	6.20	5.57	5.73	6.07
Carapace	anterior width	4.08	3.76	4.32	3.84	3.84	3.71	3.57	3.86	3.71	3.33	3.08	3.09	3.13	3.28	2.89	3.01	3.10
Carapace	posterior width	6.64	6.48	7.12	6.24	6.24	6.00	5.57	6.29	5.71	6.53	6.09	6.08	6.10	6.32	5.84	5.92	6.21
Carapace	eye diameter	0.45	0.48	0.48	0.45	0.45	0.40	0.40	0.43	0.47	0.47	0.44	0.44	0.46	0.43	0.40	0.41	0.43
Carapace	interocular distance	0.53	0.50	0.55	0.48	0.48	0.47	0.47	0.50	0.53	0.54	0.53	0.49	0.54	0.55	0.54	0.47	0.59
Carapace	ocular diada width	1.20	1.28	1.36	1.20	1.20	1.13	1.17	1.20	1.17	1.26	1.21	1.24	1.21	1.23	1.13	1.18	1.19
Tergite I	length	1.12	1.08	1.08	1.00	1.00	1.00	1.07	1.00	0.87	1.24	1.16	1.18	1.16	1.14	1.09	1.01	1.17
Tergite II	length	1.44	1.28	1.52	1.24	1.24	1.27	1.27	1.27	1.00	1.50	1.32	1.44	1.42	1.35	1.23	1.31	1.41
Tergite III	length	1.76	1.60	1.88	1.56	1.68	1.47	1.73	1.60	1.67	1.88	1.77	1.68	1.61	1.68	1.64	1.67	1.76
Tergite IV	length	2.20	2.00	2.32	2.08	2.04	1.87	2.00	2.07	1.93	2.35	2.16	2.20	2.01	2.10	1.94	2.00	2.06
Tergite V	length	2.32	2.20	2.60	2.28	2.24	2.07	2.20	2.20	2.33	2.52	2.44	2.34	2.33	2.28	2.19	2.28	2.38
Tergite VI	length	2.68	2.40	2.84	2.44	2.52	2.27	2.40	2.40	2.53	2.74	2.56	2.58	2.48	2.51	2.44	2.47	2.56
Tergite VII	length	3.88	3.96	4.20	3.80	3.84	3.60	3.80	4.07	3.80	4.60	4.09	4.06	4.05	4.11	4.01	3.83	4.28
Mesosoma	total length (tergites)	15.40	14.52	16.44	14.40	14.56	13.53	14.47	14.60	14.13	16.83	15.50	15.48	15.06	15.17	14.54	14.57	15.62
Metasoma I	length	3.55	3.55	3.75	3.55	3.55	3.30	3.50	3.60	3.50	4.00	3.67	3.63	3.66	3.71	3.49	3.56	3.84
Metasoma I	width	2.85	2.85	2.95	2.35	2.85	2.50	2.70	2.90	2.70	2.99	2.83	2.79	2.88	2.86	2.78	2.76	2.85
Metasoma I	height	2.60	2.65	2.65	2.85	2.85	2.40	2.50	2.50	2.50	2.83	2.62	2.62	2.66	2.70	2.43	2.50	2.63
Metasoma II	length	4.55	4.40	4.80	4.35	4.50	4.20	4.30	4.50	4.30	4.97	4.57	4.67	4.69	4.71	4.32	4.57	4.64
Metasoma II	width	2.75	2.70	3.05	2.20	2.75	2.40	2.60	2.70	2.60	3.02	2.75	2.82	2.84	2.89	2.69	2.72	2.77
Metasoma II	height	2.75	2.70	2.95	2.70	2.85	2.40	2.50	2.60	2.50	2.73	2.54	2.45	2.51	2.59	2.44	2.48	2.60
Metasoma III	length	5.20	5.00	5.50	4.90	5.00	4.70	4.80	5.00	4.90	5.44	5.19	5.10	5.28	5.21	4.68	4.96	5.05
Metasoma III	width	2.85	2.70	3.20	2.25	2.80	2.40	2.70	2.70	2.60	3.09	2.77	2.79	2.83	2.88	2.67	2.79	2.95
Metasoma III	height	2.90	2.60	3.00	2.85	2.85	2.40	2.40	2.50	2.60	2.81	2.53	2.54	2.60	2.63	2.52	2.50	2.58
Metasoma IV	length	5.75	5.50	6.50	5.50	5.60	5.20	5.40	5.60	5.50	6.25	5.88	5.81	5.80	5.87	5.51	5.79	5.82
Metasoma IV	width	2.85	2.75	3.25	2.25	2.85	2.50	2.60	2.80	2.60	3.10	2.77	2.77	2.87	2.87	2.68	2.77	2.86
Metasoma IV	height	2.85	2.50	3.12	2.85	2.85	2.30	2.40	2.50	2.40	2.77	2.55	2.51	2.49	2.64	2.59	2.51	2.58
Metasoma V	length	6.83	6.57	7.74	6.70	6.76	6.00	6.10	6.50	6.30	7.24	6.74	6.67	6.93	6.92	6.43	6.58	6.84
Metasoma V	width	2.93	2.60	3.25	2.86	2.93	2.30	2.50	2.60	2.40	2.91	2.57	2.58	2.59	2.63	2.49	2.57	2.59
Metasoma V	height	2.73	2.54	3.12	2.80	2.86	2.30	2.40	2.60	2.40	2.69	2.82	2.50	2.48	2.58	2.41	2.51	2.61
Metasoma	length	25.88	25.02	28.29	25.00	25.41	18.70	19.30	20.20	19.60	27.90	26.05	25.88	26.36	26.42	24.43	25.46	26.19
Telson	vesicle length	3.84	3.77	4.23	3.77	3.77	3.40	3.40	3.70	3.50	3.57	3.41	3.38	3.46	3.51	3.33	3.37	3.43
Telson	vesicle width	2.21	1.95	2.28	1.95	1.95	1.80	1.90	2.00	1.90	2.10	1.96	1.93	2.05	2.11	1.88	1.89	2.03
Telson	vesicle height	2.15	2.08	2.28	2.08	2.02	1.80	1.90	2.00	1.90	2.17	2.01	2.00	2.10	2.12	1.90	1.97	2.11
Telson	aculeus length	2.80	2.67	2.99	2.67	2.67	2.50	2.60	2.70	2.60	2.68	2.54	2.53	2.63	2.56	2.52	2.49	2.57
Telson	total length	6.24	5.92	6.76	5.98	6.05	5.80	5.50	5.90	5.70	6.80	6.31	6.50	6.71	6.61	6.21	6.26	6.41
Metasoma+ Telson	total length	32.12	30.93	35.05	30.98	31.46	29.20	29.60	31.10	30.20	34.70	32.36	32.38	33.07	33.03	30.64	31.72	32.60
Femur	length	6.18	5.98	6.70	6.18	6.11	5.60	5.80	6.00	5.90	6.96	6.46	6.51	6.76	6.72	6.39	6.41	6.61
Femur	width	1.50	1.56	1.76	1.69	1.56	1.40	1.50	1.50	1.50	1.75	1.56	1.61	1.62	1.64	1.54	1.60	1.60
Patella	length	6.76	6.70	7.28	6.44	6.57	6.00	6.00	6.20	6.30	6.68	6.10	6.12	6.54	6.31	5.78	6.01	6.21
Patella	width	2.08	2.02	2.02	2.08	2.02	1.80	1.90	2.00	1.90	2.12	1.88	1.93	2.01	2.00	1.85	1.90	1.94
Chela	length	11.50	11.10	13.00	11.57	10.50	10.40	10.60	11.20	10.90	12.26	11.28	11.25	11.75	11.77	10.79	11.08	11.63
Chela	width	2.60	2.00	2.47	2.34	2.10	1.80	2.00	2.00	1.90	2.32	2.04	1.96	2.06	2.08	1.90	2.01	2.02
Chela	height	2.10	2.10	2.73	2.21	2.10	1.80	2.00	2.10	2.00	2.10	1.81	1.85	1.92	1.95	1.84	1.82	1.93
Chela	movable finger length	8.00	7.20	8.97	7.80	7.20	7.00	7.00	7.40	7.30	8.29	7.62	7.61	8.01	8.02	7.45	7.51	7.99
Chela	fixed finger length	6.80	6.40	7.67	6.89	6.20	6.20	6.10	6.60	6.40	7.07	6.55	6.74	7.05	6.91	6.17	6.89	6.92
Chela	palm length	4.00	3.80	4.68	4.16	3.90	3.40	3.70	3.70	3.70	4.14	4.11	4.09	4.16	4.15	3.92	4.03	4.03

###### Coloration.

General pattern (in ethanol 70%) (Fig. 7): light yellow, without variegated pigmentation. **Carapace** (Figs 7A, 8A): light yellow; lateral and median eyes, surrounded by black variegated pigments. **Chelicerae** (Figs 7A, 8A): coxa and hand light yellow, without pigments; fingers, dark reddish-brown. **Mesosoma, coxosternal region, pedipalps, legs** (Fig. 7A, B): all light yellow. **Metasoma** (Fig. 7A, B): segments light yellow, progressively becoming darker towards the telson. **Telson** (Fig. 7A, B): dark yellow; aculeus dark reddish-brown. Live coloration pattern (Fig. 13A–C) similar to that of preserved specimens, except for mesosoma with a faint brown median stripe crossing all tergites, telson light reddish-brown, pedipalp chela fingers and metasomal segments IV–V dark reddish-brown.

###### Morphology.

**Carapace** (Fig. 2B): densely covered with fine granulation and few coarse granules; anterior margin with deep median notch; anterior median carinae only feebly marked over anterior 1/3; central lateral, central median, lateral ocular, posterior, posterior median and superciliary carinae, all well-marked; and furrows (anterior median, anterior marginal, central transverse, lateral ocular, supercialiary, posterior transverse, posterior lateral and posterior marginal), all well-marked; ocular tubercle well-marked, located on the anterior half of carapace; median eyes separated by about 0.53 ocular diameters; with three pairs of lateral eyes and two pairs of lateral micro-ocelli.

**Chelicerae** (Fig. 8B): dentition characteristic of the family Buthidae (Vachon 1963), densely covered with setae over the internal and ventral surfaces.

**Figure 9.**
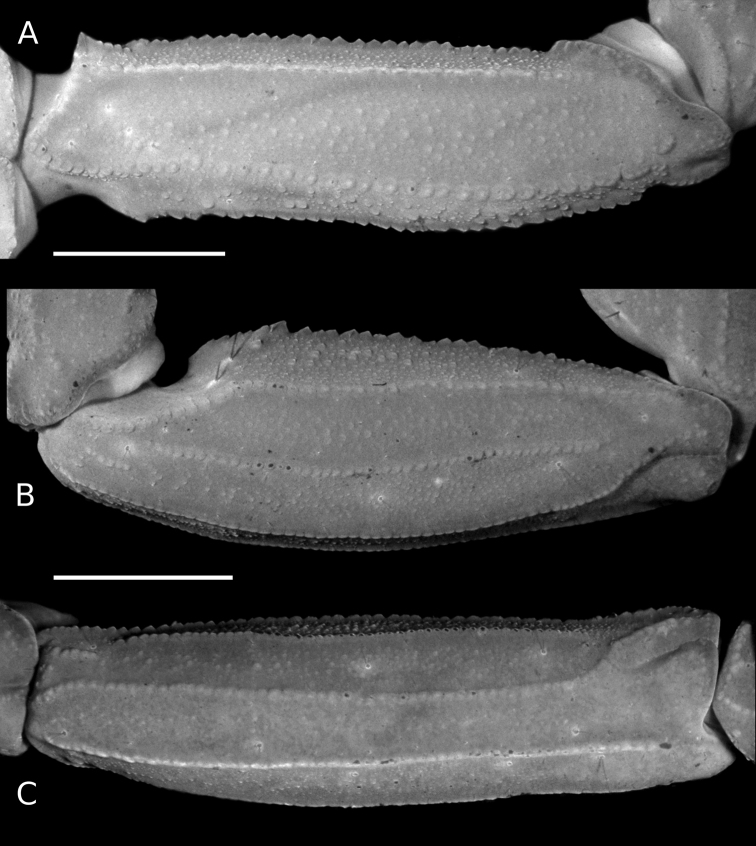
Tityus (Tityus) spelaeus sp. nov., female holotype (MZSP 74633), right pedipalp segments **A** femur, dorsal view **B, C** patella **B** dorsal view **C** external view. Scale bars: 1.5 mm.

**Figure 10.**
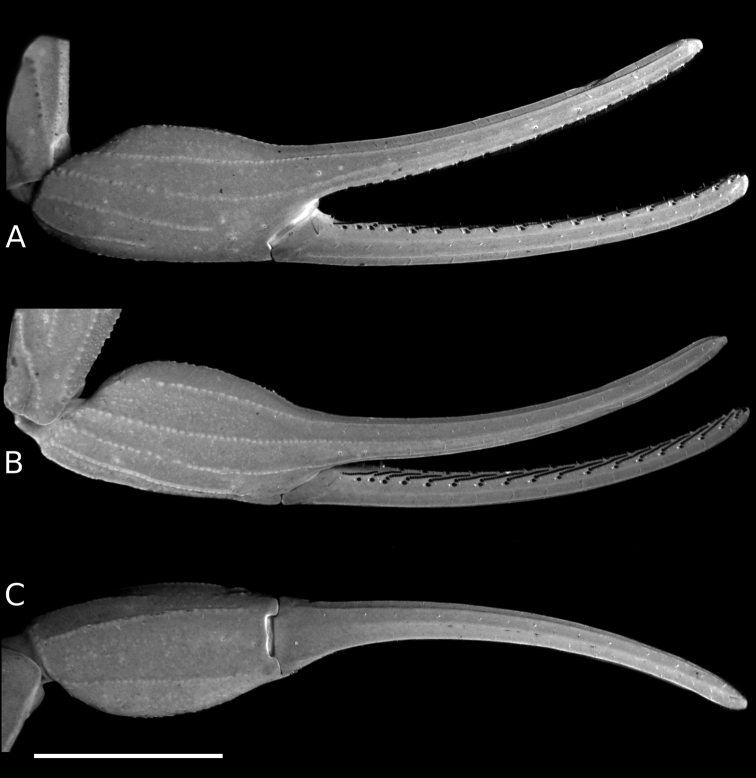
Tityus (Tityus) spelaeus sp. nov., female holotype (MZSP 74633), chela **A** external view **B** dorsal view **C** ventral view. Scale bar: 3 mm.

**Pedipalps**: Chela, short and slender (female, L/W= 5.5). Orthobothriotaxic pattern Type A, femur with alfa configuration (hand: Eb3:Eb2:Eb1:Esb:Est:Et, fixed finger: eb:esb:est:et:db:dt:it). **Femur** (Fig. 9A) with five carinae: VI, DI, DE, and VE crenulate, EM serratocrenulate, complete and pronounced, with intercarinal areas densely covered with fine granulation and few coarse granules. **Patella** (Fig. 9B, C) with seven carinae: VI, VE, DI, DE, and EM complete and crenulate; DM incomplete and crenulate; IM complete and serratocrenulate, with a short spiniform granule near the segment base; with intercarinal areas densely covered with fine granulation. **Chela** (tibia) (Fig. 10A–C) with eight carinae: VI, VE, D, DS, DMA, IM, and ES, complete and crenulate; SA, incomplete and crenulate, only present on the anterior half of the hand. Pedipalp movable and fixed fingers without basal lobe (Fig. 10A). Movable finger with 17–17 rows.

**Coxosternal region** (Fig. 7B): Sternum with posterior depression, outer ridge, and apical button, well-marked; sclerite covered with fine granulation, and few setae, except for the coxapophyses I–II, which are smooth; genital operculum longitudinally divided, composed of two sub-triangular plates.

**Figure 11.**
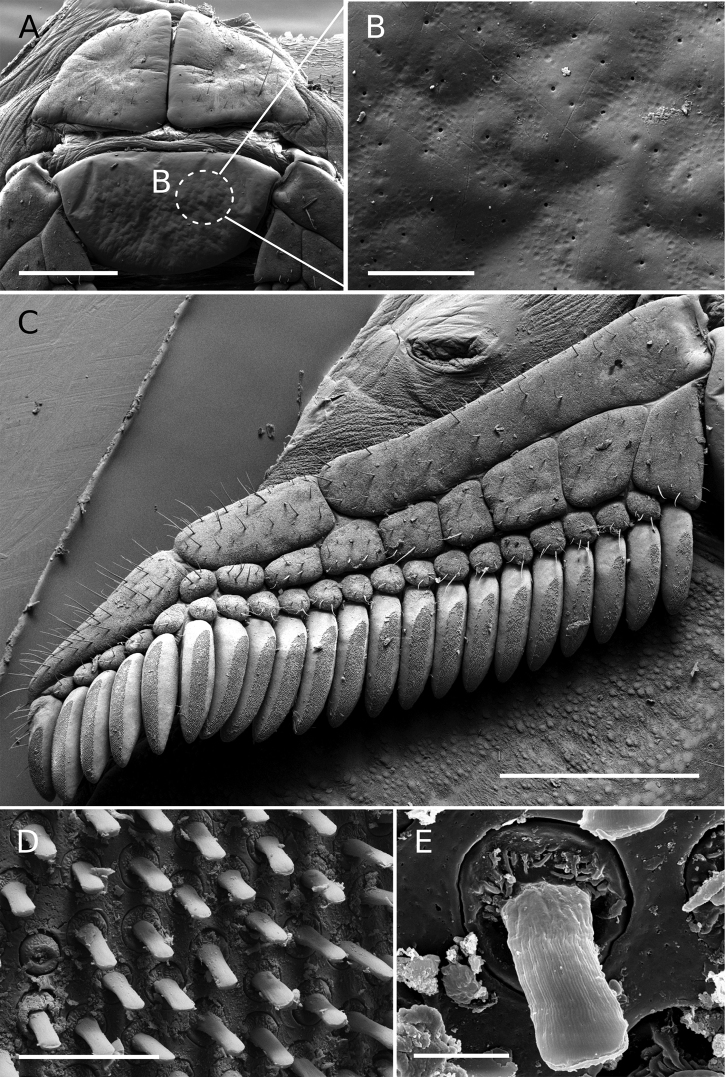
Tityus (Tityus) spelaeus sp. nov., female paratype (MZSP 74633) genital area and pectines **A** genital operculum and pectinal basal piece **B** closeup of the pectinal basal piece, showing cuticular pores on glandular region **C** right pectine **D** peg sensillae, distribution **E** closeup of a peg sensilla. Scale bars: 800 µm (**A**); 60 µm (**B**); 1000 µm (**C**); 20 µm (**D**); 3 µm (**E**).

**Pectines** (Fig. 11). Pectinal basal piece sub-rectangular and covered with a large and raised glandular region occupying beyond the anterior two thirds of the anteromedian region (Figs 6E, F, 11A, B; Table 3); pectinal tooth count of 19–22. Marginal lamellae, median lamellae, and fulcra moderately covered with setae (Fig. 5C). Basal middle lamellae, not dilated (Figs 6E, F, 11C). Pectinal tooth peg sensillae rectangular in cross-section, with a narrow distal openning (Fig. 11D, E).

**Legs**: Carinae present; intercarinal areas with sparse fine granulation; ventral telotarsal macrosetae acute and fine, arranged in two ventrosubmedian rows (Fig. 4K, L); telotarsi, counts of ventral macrosetae in the left (L) and right (R) legs on prolateral (pro) and retrolateral (retro) rows of legs I to IV (L (pro/retro) R (pro/retro)): 7/6 7/7: 7/7 7/7: 9/8 9/10: 10/10 9/11. Claws short and symmetrical.

**Mesosoma**: Tergites I–VI, moderately covered with fine granulation and few coarse granules; pre-tergites well defined, with median carina visible on the posterior margin of the post-tergites; tergite VII with DSM and DL carinae complete and crenulate, and median carina composed of a crenulate anteromedian eminence present on the anterior half of the post-tergite. Sternites densely covered with fine granulation; sternites III–VI with a pair of elliptic spiracles on the posterior half, which are progressively larger; sternite V with a hyaline subtriangular area on the posterior margin; sternite VI with VSM carinae crenulate, present on posterior half; sternite VII with VSM and VL carinae crenulate, present on posterior two thirds.

**Figure 12.**
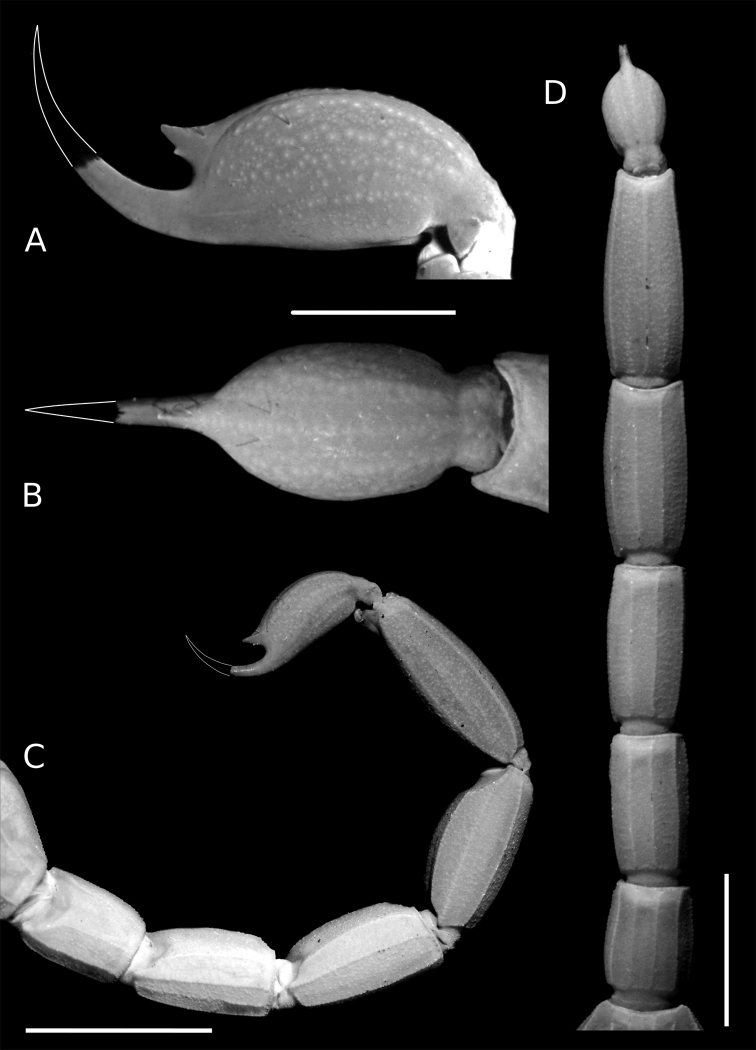
Tityus (Tityus) spelaeus sp. nov., female holotype (MZSP 74633), metasoma and telson **A, B** telson **A** lateral view **B** ventral view **C, D** metasoma **C** lateral view **D** ventral view. Scale bars: 2 mm (**A, B**); 5 mm (**C, D**).

**Metasoma** (Fig. 12C, D): Segments II–V short and robust (L/W ratio: II= 1.9; III= 1.9; IV= 2.0; V= 2.5); segment V not incrassate (Fig. 12C). Segments I–II (Fig. 12C, D) with 10 complete carinae, parallel to one another and crenulate (paired DSM, DL, ML, VL, and VSM), ML of segment II represented by coarse granules on posterior two thirds, intercarinal areas densely covered with fine granulation; segments III–IV (Fig. 12C, D) with eight complete carinae, parallel to one another and crenulate (paired DSM, DL, VL, and VSM), intercarinal areas densely covered with fine granulation; segment V (Fig. 12C, D) with five complete carinae, crenulate (VM, paired DSM, and VL: DSM carinae feebly marked), intercarinal areas moderately covered with fine granulation and few coarse granules. Segments II–IV (Fig. 12C) with DSM carinae feebly marked, composed of evenly sized granules, without enlarged distoterminal granule.

**Metasomal macrosetae**: Segments I–IV each with two pairs of VSM macrosetae (2/2): pair of VSM1 located on the anterior third, and pair of VSM2 located near posterior margin of segment; and with two pairs of VL macrosetae (2/2): pair of VL1 located near anterior margin of segment, and pair of VL2 located on posterior two thirds of segment. Segment V with two pairs of VSM macrosetae (2/2), two pairs of VL macrosetae (2/2), and a single pair of ML macrosetae (1/1); pairs of VSM1 and VL1 located near anterior margin of segment; pair of VL2 located on posterior two thirds of segment, and pair of ML1 located dorsolaterally behind the DSM carinae near posterior margin of segment; anal arch with two pairs of setae on the intercrestal area: one pair of VSM macrosetae (1/1) and one pair of VL macrosetae (1/1).

**Telson** (Fig. 12A, B): Vesicle suboval, not elongated (L/H= 1.8), dorsal surface smooth, lateral surfaces with shallow longitudinal furrow; with VM, paired VSM, VL, and DL carinae, vestigial. Subaculear tubercle large, conical, with spiniform apex directed towards the distal region of the aculeus (Fig. 12A); subaculear tubercle with ventral pair of small, rounded granules, pointing towards the basal portion of the aculeus; aculeus strongly curved, shorter than vesicle and with ventral groove.

###### Variability (females).

***Morphometrics.*** Total length (including telson): 48.30–57.98 mm (n= 17, mean= 52.70, standard deviation (SD)= 2.66). Chela L/W ratio: 4.42–5.78 (n= 17, mean= 5.44, SD= 0.37). Metasomal segment I L/W ratio: 1.24–1.51 (n= 17, mean= 1.30, SD= 0.06). Metasomal segment V L/W ratio: 2.31–2.68 (n= 17, mean 2.52, SD= 0.12). Telson vesicle L/H: 1.63–1.89 (n= 17, mean= 1.76, SD= 0.09). ***Meristics*.** Pectinal tooth count: 19–22 (n= 34, mode= 20). Number of movable finger oblique granular rows: 16–18 (n= 34, mode= 18). Metasomal macrosetae count: (n= 17): 2/2 VSM and 2/2 VL macrosetae on segments I–IV, 3/3 VSM and 2/2 VL macrosetae on segment V. However, one specimen (LES/UFSCar 14668) lost VSM1 on segment II, a second specimen (LES/UFSCAR 14669) lost VL1 on segment II, and a third specimen (LES/UFSCAR 014673) lost one VSM1 on segment I. Variation in the count of telotarsal ventrosubmedian setae is presented in Table 5.

**Table 5.** Variation in the number of macrosetae of the ventrosubmedian setal rows on telotarsi I–IV across paratypes of *Tityusspelaeus* sp. nov. Abbreviations: L, left leg; Pl, prolateral row; Rl, retrolateral row; R, right leg.

**Telotarsus**	**MZSP 74634 (1)**		**MZSP 74634 (2)**		**MZSP 74634 (3)**		**MZSP 74634 (4)**	
	**L(Pl/Rl)**	**R(Pl/Rl)**	**L(Pl/Rl)**	**R(Pl/Rl)**	**L(Pl/Rl)**	**R(Pl/Rl)**	**L(Pl/Rl)**	**R(Pl/Rl)**
**I**	8/7	8/7	6/7	8/6	8/7	7/8	6/7	6/7
**II**	7/8	8/7	8/8	8/9	8/8	7/8	8/8	8/7
**III**	8/8	7/8	8/7	8/9	10/8	8/9	6/8	8/7
**IV**	10/10	10/10	10/10	-	9/12	10/9	10/10	10/11
**Telotarsus**	**MZSP 52228**		**MZSP 52230**		**MZSP 52229**		**MZSP 52231**	
	**L(Pl/Rl)**	**R(Pl/Rl)**	**L(Pl/Rl)**	**R(Pl/Rl)**	**L(Pl/Rl)**	**R(Pl/Rl)**	**L(Pl/Rl)**	**R(Pl/Rl)**
**I**	6/6	-	7/8	7/6	7/6	7/7	6/7	6/8
**II**	7/7	7/8	7/7	8/7	9/6	7/7	8/7	7/8
**III**	9/7	8/8	7/8	8/7	7/6	10/8	6/6	8/8
**IV**	-	10/12	10/11	9/8	10/10	11/10	10/8	10/10
**Telotarsus**	**LES 14668**		**LES 14669**		**LES 14670**		**LES 14671**	
	**L(Pl/Rl)**	**R(Pl/Rl)**	**L(Pl/Rl)**	**R(Pl/Rl)**	**L(Pl/Rl)**	**R(Pl/Rl)**	**L(Pl/Rl)**	**R(Pl/Rl)**
**I**	8/7	8/7	8/9	8/8	9/8	6/7	8/8	9/9
**II**	8/7	8/7	10/9	9/8	9/8	10/7	9/9	8/8
**III**	8/8	9/8	9/10	9/9	9/8	8/8	9/9	9/9
**IV**	11/11	11/10	10/11	10/11	10/10	10/10	12/12	10/11
**Telotarsus**	**LES 14672**		**LES 14673-1**		**LES 14673-2**		**LES 14673-3**	
	**L(Pl/Rl)**	**R(Pl/Rl)**	**L(Pl/Rl)**	**R(Pl/Rl)**	**L(Pl/Rl)**	**R(Pl/Rl)**	**L(Pl/Rl)**	**R(Pl/Rl)**
**I**	9/8	8/8	8/8	8/8	8/9	9/8	8/8	9/9
**II**	9/8	8/8	8/9	8/8	10/9	9/8	8/8	8/8
**III**	9/9	10/9	9/9	8/8	9/9	10/10	9/9	9/8
**IV**	10/11	11/12	11/10	11/10	12/12	11/12	12/11	11/12

###### Natural history.

Russão II cave is formed by limestone (a karstified type of rock), located in Posse municipality, the northeastern state of Goiás, Central Brazil. This karst region is part of the Bambuí geomorphological group, the large geomorphological group in Brazil, occurring in states of Bahia, Goiás, Minas Gerais, and Tocantins. Russão II cave is inserted on the Cerrado morphoclimatic domain (Ab’Saber 1977), and the climate is tropical semi-humid (Nimer 1979). There is a stream crossing the cave although there are no surface drainages nearby (Tencatt and Bichuette 2017). Russão II cave is located on private property, and in addition surface habitats are under impact from pollution through the discharge of domestic sewage, deforestation of surroundings for cattle pasture, and small mining projects (Tencatt and Bichuette 2017). Russão II cave, like other caves in the region, has no legal protection under Brazilian environmental laws. The cave has a significant amount of bat guano piles and a large cricket population that is preyed upon by scorpions. In the aphotic zone of Russão II cave, the temperature was 30.04 °C, the relative humidity of the air was 72.02%.

**Figure 13.**
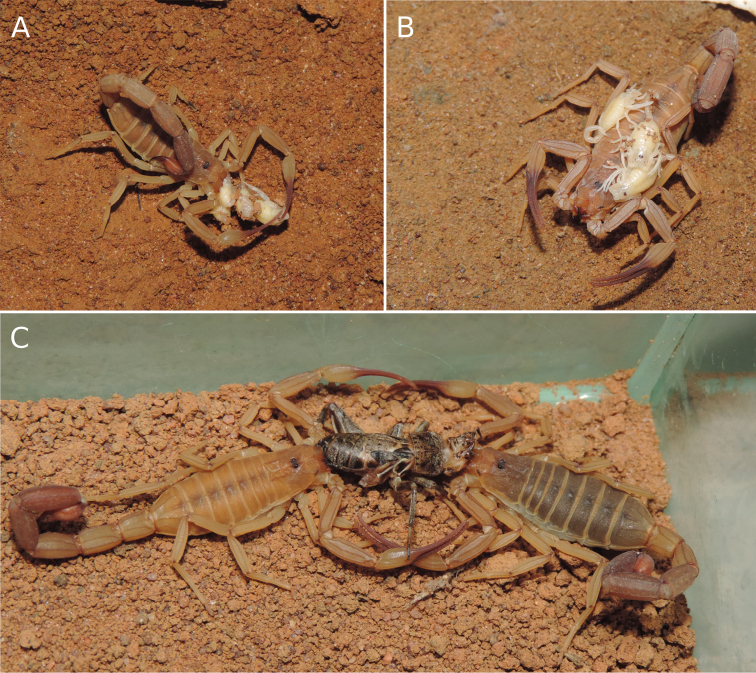
Tityus (Tityus) spelaeus sp. nov., female paratypes under laboratory conditions **A, B** female paratype with scorpionlings **A** feeding upon scorpionlings **B** litter on female’s back **C** specimens feeding on a cricket.

**Figure 14.**
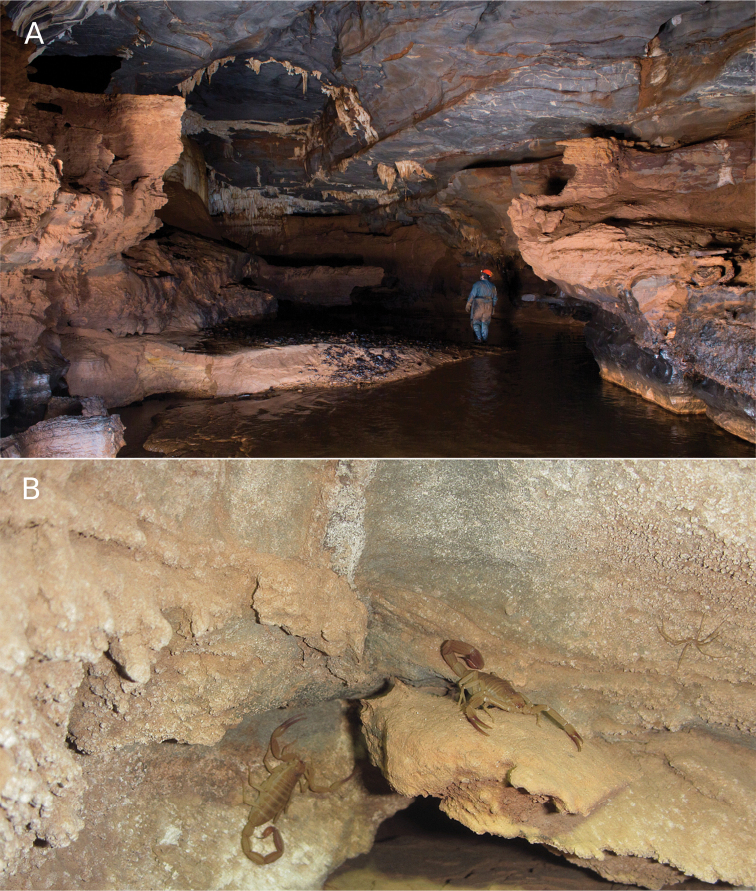
Habitat of Tityus (Tityus) spelaeus sp. nov. in the Russão cave **A** inside landscape of the cave **B** females on the cave walls.

This species was studied in the past by Outeda-Jorge et al. (2009) who reported a litter size of two scorpionlings, but under laboratory conditions (Fig. 13A–C), another two females had a litter of four scorpionlings, and both females were fed upon their litter (Fig. 13A, B). The population of *Tityusspelaeus* sp. nov. at the Russão cave is well-established (Fig. 8A, B). During a one-hour-long visit to the cave in 2007, more than 20 live scorpions were observed on the ground and walls (Fig. 14A, B). In another two-hour visit in 2015, 32 individuals were counted, both adults and juveniles.

## ﻿Discussion

### ﻿Phylogenetic patterns within *Tityus*

Our phylogenetic results (Figs 1–3) are similar to those of previous studies (i.e., Ojanguren-Affilastro et al. 2017a). In our analysis using molecular evidence, we found that Tityus (Tityus) is polyphyletic and Tityus (Atreus) is paraphyletic (Figs 1–3). This agrees with previously published *Tityus* phylogenies, which found discrepancies in the subgeneric classification of the genus proposed by Lourenço (2006). In the molecular phylogeny of Ojanguren-Affilastro et al. (2017a), which incorporated 18 *Tityus* terminals representing three subgenera, Tityus (Tityus) was found to be polyphyletic, with one clade containing *T.bahiensis*, *T.stigmurus*, and *T.trivittatus* species-groups as the sister group of Tityus (Archaeotityus), and another clade consisting of the *T.bolivianus* species-group as the sister group of Tityus (Atreus). Our results are highly consistent with those results (Figs 1–3), since we also recovered Tityus (Tityus) (here including *T.brazilae*) as the sister group of Tityus (Archaeotityus), and Tityus (Atreus) (here including *T.sastrei*) as the sister group of the *T.bolivianus* species-group [referred to as Tityus (Tityus)- *T.bolivianus* species-group in Ojanguren-Affilastro et al. (2017a)].

More recently, Román et al. (2018) analyzed 51 terminals of 26 species [including 22 Tityus (Atreus) species] and recovered the *T.obscurus* species-group as paraphyletic. However, their study had a problem, because Román et al. (2018) mixed up members of the *T.androcottoides* species-group with members of the *T.obscurus* species-group. In reality, Román et al. (2018) recovered a monophyletic *T.obscurus* species-group and a polyphyletic *T.androcottoides* species-group (mostly composed of Venezuelan species). In our hypothesis, Tityus (Atreus) was recovered as monophyletic upon transferal of Tityus (Tityus) sastrei to Tityus (Atreus) (Figs 1–3).

Finally, other aspects were not challenged by several authors, such as the consensus about Tityus (Archaeotityus) (i.e., *T.clathratus* species-group) being the sister clade of the remaining groups of *Tityus*. Indeed, this notion was discussed and supported by several authors during the last 75 years (Mello-Leitão 1945; Lourenço 1999, 2002a, 2002b; Borges et al. 2010). Some have assumed without any phylogenetic support that the small size, large subaculear tubercle, and cryptic coloration patterns of Tityus (Archaeotityus) scorpions are plesiomorphic character states (e.g., Lourenço 1999). Nevertheless, the phylogenetic analysis of Ojanguren-Affilastro et al. (2017a) recovered Tityus (Archaeotityus) as the sister group of a clade composed of some Tityus (Tityus) terminals, not the sister group of all *Tityus* subgenera, something that we also recovered in our results (Figs 1–3). Likewise, Borges et al. (2012) found a close relationship between the toxin composition of the *T.clathratus* and *T.stigmurus* species-groups. Based on a comprehensive phylogenetic analysis of Tityus (Archaeotityus) carried (Moreno-González 2021) and the results of this investigation, Tityus (Archaeotityus) cannot be considered as the sister clade of other *Tityus* subgenera or species-groups, as previously believed.

### ﻿On the position of *Tityusbrazilae* Lourenço & Eickstedt, 1984

In the original description of *Tityusbrazilae* Lourenço & Eickstedt, 1984 the species was associated with *Tityuscostatus* (Karsch, 1879) (referred to as *Tityusdorsomaculatus* Lutz & Mello, 1922) mainly due to the similar coloration pattern of the body of both species (Lourenço and Eickstedt 1984). However, Lourenço and Eickstedt (1984) also mentioned that the slender and elongated male pedipalp of *T.brazilae* is very common among some Amazonian species such as *Tityusobscurus* Pocock, 1897. Lourenço (2002b: 167) argued: “The fact I have included *Tityusbrazilae* in the *Tityusasthenes* species-group, may surprise some readers because this species presents a pattern of pigmentation which excludes it from the group of dark or blackish scorpions. I based my decision, however, on the general morphology of the species and on the type of sexual dimorphism it displays.” Lourenço (2006) ended up including *Tityusbraziliae* in the subgenus Tityus (Atreus).

It is worth mentioning that the slender and elongated shape of the male pedipalp has been demonstrated to be a highly homoplastic character state that evolved independently at least four times within *Tityus* (Moreno-González, 2021). For this reason, the shape of the male pedipalp must be used with caution and used in conjunction with other morphological characters and molecular data, such as those proposed in this paper, to correctly classify *Tityus* species into species-groups.

For example, the position of *Tityusbrazilae* into the *Tityusobscurus* species-group (previously the *Tityusasthenes* species-group) of the subgenus Tityus (Atreus) is contradicted by our molecular and morphological evidence (e.g., Figs 1–3). In fact, *Tityusbrazilae* exhibits some character states shared by all members of the *Tityusbahiensis* species-group of the subgenus Tityus (Tityus) (plus all the members of the *Tityusstigmurus* and *Tityustrivittatus* species-groups) such as: **i)** ventral macrosetae of telotarsi I–IV distributed in two ventrosubmedian rows (type II) (Fig. 4A, B), **ii)** basal middle lamellae of female pectines not dilated and without glandular regions (Fig. 5A, B), and **iii)** female pectinal basal piece with a well-developed glandular region (Fig. 5A, B). In contrast, all the members of the *Tityusobscurus* species-group exhibit: **i)** telotarsi I–IV ventral macrosetae irregularly distributed in a tuft (type I) (Fig. 4G, H), **ii)** basal middle lamellae of female pectines dilated (subcircular) and with glandular regions (Fig. 6A ,B), and **iii)** female pectinal basal piece without glandular region whatsoever (Fig. 6A, B).

### ﻿On the position of the *Tityusbolivianus* species-group and *Tityussastrei* Lourenço & Flórez, 1990

Lourenço (2006) assigned all the species of the *Tityusbolivianus* species-group and the species *Tityussastrei* to the subgenus Tityus (Tityus). This decision was based on a combination of morphological characters that, according to Lourenço (2006), allow the diagnosis of Tityus (Tityus): **i)** total body length between 50–80 mm, **ii)** coloration pattern pale yellow to dark brownish frequently with confluent or longitudinal spots, **iii)** pectines with 15–26 teeth, **iv)** movable finger with 15–18 dorsal oblique rows of granules, and **v)** subaculear tubercle frequently acute.

Given the results of our phylogenetic analysis, previous hypotheses (Ojanguren-Affilastro et al. 2017a), and the phenotypic characters explored in this paper (i.e., the ventral macrosetae of telotarsi, the female pectinal piece glands, and the basal middle lamellae of female pectines), it seems that the *Tityusbolivianus* species-group and *Tityussastrei* are not part of Tityus (Tityus) (Figs 2, 3). According to our observations, Tityus (Tityus) presents well-developed glandular areas in the pectinal basal piece of females (e.g., Figs 3, 5A, B, C, D, 6C, D, E, F; Table 3) and do not exhibit dilation and glandular region in the basal middle lamellae of female pectines (e.g., Figs 3, 5A, B, C, D, 6C, D, E, F; Table 3). Whereas, in terminals such as members of the *Tityusbolivianus* group and in *Tityussastrei*, glandular areas are absent in the pectinal basal piece of females (e.g., Fig. 3; Table 3) and the basal middle lamellae of female pectines are always dilated and exhibit glandular areas (Table 3). Consequently, *Tityussastrei* was transferred to Tityus (Atreus), whereas the *Tityusbolivianus* species-group awaits for an appropriate subgeneric designation based on a broader phylogenetic analysis of *Tityus* (i.e., Moreno-Gonzalez, 2021)

### ﻿Phenotypic characters

#### Distribution of ventral setae of telotarsi I–IV

The leg telotarsi ventral setation has been a very commonly used phenotypic character to define genera and/or assist species diagnoses in families such as Bothriuridae, Chactidae, Diplocentridae, and Vaejovidae (e.g., Lourenço 2002a, 2002b; Prendini 2003b; Mcwest 2009). However, it has been a neglected morphological character in the taxonomy of all the New World buthid genera, including *Tityus*. For instance, after being used in an identification key of *Tityus* species presented by Kraepelin (1895), the distribution of the ventral macrosetae of the telotarsi was never again used for species identification. In fact, very few descriptions of *Tityus* species have described the distribution of the ventral macrosetae of telotarsi I–IV (e.g., Ojanguren-Affilastro et al. 2017b), and none have implemented existing interspecific variations into modern taxonomic diagnoses or identification keys.

It is particularly interesting to note that the *Tityus* species that have ventral setae tufts on the telotarsi, for instance members of the Tityus (Archaeotityus) or the *T.obscurus* species-group of Tityus (Atreus), tend to be more strongly associated with vegetation and trees, and some are more prone to climb up to the top of the trees. On the contrary, species with two ventrosubmedian rows of setae, for instance some members of Tityus (Atreus), such as species in the *T.forcipula* species-group and *T.sastrei*, have a stronger association with bark, lower vegetation, rotten logs, and soil in general, but not with the canopy. However, after a SEM survey of the ventral setal distribution of telotarsi I–IV across different species of *Tityus* (Moreno-González, 2021), no significant differences were found in the ultrastructure of the setae from tufts (type I) or the ventrosubmedian rows (type II). Both setae have a striated surface and no other obvious modifications, much like setae from other body regions.

This previously ignored morphological character has sometimes been proved useful to assist taxonomic delimitations, even outside the genus *Tityus*. For example, Esposito et al. (2017, 2018) included the ventral setae of telotarsi in their morphological matrix, although they did not use it to assist the diagnoses of Centruroidinae genera. However, according to our observations, the distribution of the ventral macrosetae of telotarsi do not significantly vary between legs or species of the same species-group, nor are these sex- or maturity dependent, thus representing an informative characters for the recognition of Centruroidinae genera: type I in *Centruroides* Marx, 1890, *Physoctonus* Mello-Leitão, 1934, and *Rhopalurus* Thorrell, 1876; type II in *Heteroctenus* Pocock, 1893, *Jaguajir* Esposito, Yamaguti, Souza, Pinto-da-Rocha & Prendini, 2017, *Ischnotelson* Esposito, Yamaguti, Souza, Pinto-da-Rocha & Prendini, 2017, and *Troglorhopalurus* Lourenço, Baptista & Giupponi, 2004. For this reason, we consider it is important to incorporate this character into the diagnoses of New World buthid taxa.

#### The basal piece and basal middle lamellae of the female pectines

The sexual dimorphism of the basal pectinal piece and the glands that it sometimes carries are characters that have been neglected in the taxonomy of *Tityus*. Here we continued the exploration of the pectinal piece morphology started by Moreno-González et al. (2019), including additional species-groups and subgenera of *Tityus*.

The glandular region of the pectinal basal piece of female has far too often been an overlooked morphological character in taxonomic and systematic contributions dealing with buthid taxa. Moreno-González et al. (2019) suggested, for the first time, that the presence of a glandular region on the pectinal basal piece of female is a useful character for the recognition of Tityus (Archaeotityus). In the present contribution, we discovered that the evaluation of the morphology of the pectinal basal piece of females helps make taxonomic decisions at the species and species-group levels. In the analyzed terminals of *Tityus*, we detected four character states for the presence and development of the glandular region on the basal piece (see Results). Those character states were very congruent with the topology (Fig. 3) (i.e., Moreno-González 2021). For this reason, we consider that the pectinal basal piece provides valuable information, and we urge all incoming species descriptions to incorporate a detailed description of this structure and to use it in the construction of comparative taxonomic diagnoses when relevant.

On the other hand, the dilatation exhibited by the basal middle lamellae of the female pectines has been a widely used character in the taxonomy of *Tityus* (e.g., Lourenço 2000, 2002a, 2002b). It is worth noting that, when these lamellae are dilated, there is no glandular region in the pectinal basal piece, except in the *T.androcottoides* species-group of Tityus (Atreus). In this group, the basal pectinal piece may bear a glandular region in both sexes (e.g., *T.rebierei*), something not previously reported in any other study. It is possible that these glandular regions could play a crucial role in chemical communication, but specific studies are needed to evaluate this hypothesis.

Cuticular (exocrine) glandular regions are a very common feature in a broad spectrum of arthropod groups (e.g., Coleoptera, Hemiptera, Hymenoptera, Isoptera, Lepidoptera, and Orthoptera) (Costa-Leonardo et al. 2009; Schiestl 2010; Richard and Hunt 2013; Pelosi et al. 2014; Blomquist et al. 2020). However, in *Tityus* species, the glandular function of these regions, present on the pectinal basal plate and basal middle lamellae of the female pectines, and the sternites of both sexes, remains unexplored. Nevertheless, all these regions exhibit a high density of cuticular pores when compared to other body parts (e.g., Fig. 11A, B), which leads us to think that they may secrete chemicals. But again, more studies are required to corroborate this hypothesis.

#### On cave-dwelling scorpions from Brazil

Species of two scorpion families occur in Brazilian caves, Bothriuridae Simon, 1880 and Buthidae (Trajano 1987; Trajano and Moreira 1991; Gnaspini and Trajano 1994; Pinto-da-Rocha 1995; Cordeiro et al. 2014). Few specimens of Bothriuridae have been recorded in Brazilian caves, with *Bothriurusaraguayae* Vellard 1934 having been recorded from caves in the states of São Paulo (Iporanga municipality) and Minas Gerais (Itacarambi municipality), and *Thestylusaurantiurus* Yamaguti and Pinto-da-Rocha, 2003 from one granitic cave in the state of São Paulo (Bichuette et al. 2017). Considering their burrowing habits, coupled with the few records in caves, Bothiuridae species probably are accidental fauna in subterranean habitats.

Representatives of Buthidae are more found in Brazilian caves, with at least eight species having been recorded, belonging to the genera *Tityus* and *Troglorhopalurus* (Lourenço et al. 1997; Esposito et al. 2017). One undoubtedly troglobitic species, *Troglorhopalurustranslucidus* Lourenço, Baptista and Giupponi 2004, is known from sandstone caves in Chapada Diamantina, state of Bahia. Two other species are probably accidental, Tityus (Atreus) obscurus and *Ischnotelsonperuassu* Esposito, Yamaguti, Souza, Pinto-da-Rocha and Prendini 2017, each with records from caves in the states of Pará (Altamira region) and Minas Gerais (Itacarambi region), respectively (authors, pers. obs.). Some other species are troglophiles, such as Tityus (Tityus) blaseri which lives in caves and epigean habitats in the state of Goiás, Tityus (Tityus) confluens Borelli 1899 in caves and epigean habitats in the states of Mato Grosso and Mato Grosso do Sul, Tityus (Tityus) stigmurus (Thorell 1876) which is widely distributed in northeastern Brazil with facultative cave populations in the state of Sergipe and the new species here described, Tityus (Tityus) spelaeus sp. nov. The biospeological classification of *Troglorhopaluruslacrau* (Lourenco and Pinto-da-Rocha 1997) remains contentious due to it having cave populations in the state of Bahia and one epigean record (of its junior synonym *Rhopalurusbrejo* Lourenço, 2014) from Crato in the state of Ceará (Esposito et al. 2017). Based on those records, Esposito et al. (2017) classified *Troglorhopaluruslacrau* as a troglophile, a classification also followed by Prendini et al (2021).

As expected, troglophilic populations are found more often inside caves than in epigean habitats due to differences in the dynamics of species. They are generally more numerous in subterranean habitats (Trajano and Carvalho 2017) and, for that reason, collecting in epigean habitats to find troglophilic populations with low densities on the surface is advisable (Trajano and Carvalho 2017).

Troglophiles and trogloxenes are both found in epigean and subterranean environments, and, since individuals can move between them, it is not easy to distinguish between these two categories. One strong piece of evidence for troglophilic populations is the presence of individuals of all ages distributed along with the subterranean environment throughout different annual cycles (Bichuette and Trajano 2006; Trajano and Carvalho 2017). In both visits to Russão II cave (2007 and 2015), we found individuals of *T.spelaeus* sp. nov. of different ages, including juveniles of the second instar and pregnant females, distributed in all terrestrial zones of the cave, which signal that the new species is a troglophile.

No individuals of *T.spelaeus* sp. nov. have been found in the epigean habitat to date. However, *Tityusspelaeus* sp. nov., does not show any troglomorphisms, such as elongated appendices, reduction of visual organs, low degree of sclerotization or depigmentation. The use of clues like troglomorphisms to assume that a species is troglobitic become valid when analyzed within a phylogenetic framework, which can show that these features are autapomorphic states of troglobites (Trajano and Carvalho 2017). So, we believe that the new scorpion described here is a troglophile, and it is noteworthy that the surroundings of the Russão II cave are severely modified for cattle pastures and urban growth (Tencatt and Bichuette 2017).

Also, it is worth mentioning that troglophiles are not less adapted to subterranean environment than troglobites in what is considered a continuum of cave adaptation (Trajano and Carvalho 2017), just as troglobites do not represent an evolutionary dead-end, with some known cases of endogenous scorpions having evolved from troglobitic ancestors (Prendini et al. 2010).

## Supplementary Material

XML Treatment for Tityus

XML Treatment for Tityus (Tityus)

XML Treatment for Tityus spelaeus

## ﻿Appendix 1

Voucher samples from which material was examined for morphological study and Sanger loci were sequenced. The vouchers are deposited in the following collections: the Ambrose Monell Cryocollection (**AMCC**) of the American Museum of Natural History (**AMNH**), New York (curator: Dr. Lorenzo Prendini); the Instituto de Biociências, Arachnology Laboratory Cryo-Collection (**IBALCC**) (curator: Dr. Ricardo Pinto da Rocha); the Museum National d’Histoire Naturelle (**MNHN**), Paris, France (curator: Dr. Mark Judson); the Museu Nacional/ Universidade Federal do Rio de Janeiro (**MNRJ**), Rio de Janeiro, Brazil (curator: Dr. Adriano B. Kury); the Museu de Zoologia da Universidade de São Paulo (**MZSP**), São Paulo, Brazil (curator: Dr. Ricardo Pinto da Rocha); the Museu de aracnología, Universidade Federal de Minas Gerais (**UFMG**), Belo Horizonte, Brazil (curator: Dr. Adalberto Santos).

***Isometrusmaculatus* (DeGeer, 1778)**: Brazil: adult male, without locality data, xii.1954 (MZSP 87742). State of Pará: adult male, Belém, 1984 (MNRJ 7041); adult female, Belém-Brasilia highway km 91, 15.viii-20.x.1959 (MZSP 8743). SRI LANKA: adult male and adult female, Wellawaya, 24.ii.2000, D. Huber (AMCC [LP 1798])

***Tityusannae* Lourenço, 1997**: Brazil, state of Paraiba/ Pernambuco: adult female (holotype), 1895, Gounelle (MNHN-RS-0818).

***Tityusargentinus* Borelli, 1899**: Argentina, Salta province: two adult females, Calilegua National Park- Águas Negras section [Parque Nacional de Calilegua- Seccional Águas Negras], 23°45'38.16"S, 64°51'0.79"W, 7.xii.2008, A. Ojanguren-Affilastro & C. I. Mattoni (UFMG 15906).

***Tityusblaseri* Mello-Leitão, 1931**: Brazil, state of Goiás: subadult female (holotype), Veadeiros, Rio São Miguel, 11.ii.1882, Blaser (MNRJ 11282); adult female, Alto Paraíso de Goiás, entrance Cristal waterfall [Entrada Cachoeira Cristal], 14°05.583'S, 47°30.547'W, 5.iv.2009, F. Marques & S. Outeda-Jorge (IBALCC-RPDR 00027); subadult female, same data (IBALCC-RPDR 00114/ MZSP 31125).

***Tityusbahiensis* (Perty, 1833)**: Brazil, state of Minas Gerais: adult male and subadult female, Serra do Rola Moça National Park [Parque Estadual Serra do Rola Moça], 20°5"S, 44°2"W, 2.xii.2004, A. A. Azevedo (UFMG 4076); adult female, RPPN Cachoeira Cerradão, São Roque de Minas, -20.22797, -46.3869, 2.v.2014, R. Pinto-da-Rocha & F. Marques (IBALCC-RPDR 00281).

***Tityusblaseri* Mello-Leitão, 1931**: Brazil, state of Goiás: adult female, Alto Paraíso de Goiás, pathway to Cristal waterfall [cachoeira Cristal], 05.iv.2009, S. Outeda-Jorge & F. Marques (IBALCC-RPDR 00027, 00114).

***Tityusbraziliae* Lourenço & Eickstedt, 1984**: Brazil, state of Pernambuco: adult female, Engenho Água, Serra dos Mascarenhas, 07°36'S, 35°23'W, 24–25.vii.2010, M. B. da Silva & A. M. Souza (MZSP 75619); adult male, Goiana, 29.v.2008, H. Yamaguti, T. Porto & M. B. da Silva (IBALCC-RPDR 00199). State of Sergipe: adult male, Serra de Itabaina National Park [Parque Nacional Serra de Itabaina], 10°45'07"S, 37°20'27"W, 28.vi.2009, R. Pinto-da-Rocha (IBALCC-RPDR 00169).

***Tityuscarrilloi* Ojanguren-Affilastro, 2021**: Paraguay: two males and three females, Asunción, xi.1944 (MZSP 21772).

***Tityuscharreyroni* Mello-Leitão, 1933**: Brazil, state of Goiás: subadult female, Catalão, 12°11.755'S, 47°57.189'W, 4.iv.2009, S. Outeda-Jorge & F. Marques (IBALCC-RPDR 00112); adult female, Piranhas, 20.iv.2008 (UFMT 00340). State of Mato Grosso: adult female and four juveniles, urban area, Pontal do Araguia, 27.iv.2007, Neivander (UFMT 00343); adult female, same locality data, 14.v.2007, Neivander (UFMT 00338); adult female, João de Barro, Torixoreu, 2010, Silvana (UFMT 00341).

***Tityusclathratus* C. L. Koch, 1844**: Brazil, state of Roraima: adult male and 10 adult females, Alto Alegre, 3°00'10"N, 61°18'08"W, 10.xi.2008, H. Yamaguti & R. Pinto-da-Rocha (MZSP 31468); adult female, Amajari, Vila Tepequém, 11.xi.2008, H. Yamaguti & R. Pinto-da-Rocha (IBALCC-RPDR 00192).

***Tityusconfluens* Borelli, 1899**: Brazil, state of Mato Grosso do Sul: adult female, Gruta Pitangueiras, Bonito, 22.x.2002, E. Trajano et al., pitfall- 40 m away from the entrance (MZSP 23943).

***Tityuscostatus* (Karsch, 1879)**: Brazil, state of Minas Gerais: adult female, Fazenda Montes Claros, 19°47'S, 42°8'W, iv.2001, W. J. Cassimiro (UFMG 4088); adult female, same locality, 18.xi.2000, W. J. Cassimiro (UFMG 4077); adult female, same locality data, 18.ix.1999, W. P. Martins (UFMG 4081). State of Espírito Santo: adult male, Biological Reserve Córrego do Veado [Reserva Biologica Córrego do Veado], 18°21.280'S, 40°08.165'W, 13.vi.2011, H. Yamaguti et al. Leg., pitfall (MZSP 42883). State of Rio de Janeiro: adult female, Mangaratiba, viii.2017, D. Álvarez (MZSP).

***Tityusforcipula* (Gervais, 1843)**: Colombia, Risaralda department: three adult males and two adult females, Santuario, San Rafael Plains Natural Regional Park (Planes de San Rafael), 5°7'34"N, 76°0'26.4"W, 2158 m a.s.l., 17.x.2012, J. A. Moreno (MZSP). Valle del Cauca department: adult female, Yumbo, Dapa, Bocatoma del Acueducto, 17–18.viii.2016, J. A. Moreno (IBALCC-RPDR 00256).

***Tityusgasci* Lourenço, 1981**: French Guiana: adult male (holotype), South of French Guiana, 1975, J. P. Gasci (MNHN-RS-7921). Tityus nelsoni Lourenço, 2005: BRAZIL, Amazonas state: adult female (paratype) and adult male (holotype), São Gabriel da Cachoeira, 5–30.iii.1992, E. Soares (MNHN-RS-8619, MNHN-RS-8618).

***Tityusobscurus* Gervais, 1843**: Brazil: state of Pará: three adult males and two adult females, posto 8- sismografo, Altamira, 14.iv.2009 (MNRJ 07610). state of Amapá: juvenile, Oiapoque–Tumucumaque, Saur Maripa, 17.iii.2015, D. Chirivi & J. Murienne (IBALCC-RPDR 00236).

***Tityuspanguana* Kovařík, Teruel, Lowe & Friedrich, 2015.** Peru, Madre de Dios department: adult male, Erika Lodge, Rio Alto, 30 min on boat from Atoleya, 7–8.xii.2004, J. A. Ochoa (IBALCC-RPDR 00268).

***Tityuspintodarochai* Lourenço, 2005**: Brazil, state of Paraná: adult female (holotype), Vilha Velha National Park [Parque Estadual de Vilha Velha], 28.i.1973, J. Garzoni (MNHN-RS-6567).

***Tityuspotameis* Lourenço & Giupponi, 2004.** Brazil, state of Espírito Santo: adult female, Sooretama Biological Reserve [Reserva Biologica Sooretama], trilha da sede, 19°03'23.5"S, 40°08'51.7"W, 02.vi.2011, H. Yamaguti (IBALCC-RPDR 00275).

***Tityusraquelae* Lourenço, 1987**: BRAZIL, state of Pará: adult male and adult female (paratypes), Tefé, Mathan (MNHN-RS-0825).

***Tityusrionegrensis* Lourenço, 2006**: Brazil, state of Amazonas: adult male (holotype), between São Gabriel da Cachoeira and ´Pico da Neblina´, Rio Negro region, ii.1970, Rain Forest, in canopy, J. Lacroix (MNHN-RS-8643).

***Tityussastrei* Lourenço & Flórez, 1990**. Colombia, Valle del Cauca department: adult female, Buenaventura, vía al mar, Pericos Natural Reserve [Reserva Natural Pericos], 8.xii.2018, J. A. Moreno & N. Herreño (IBALCC-RPDR 00382).

***Tityusserrulatus* Lutz & Melo, 1922**: Brazil, state of Bahía: two adult females, between Mucugé and Igatu, 22.i.2007, C. Mattoni, R. Pinto-da-Rocha & H. Yamaguti (MZSP 28205). State of Minas Gerais: adult male, Cavernas do Peruaçu National Park [Parque Nacional Cavernas do Peruaçu], Januária, -15.12383, -44.24111, 4–25.i.2009, R. S. Recoder & M. Teixeira Jr. (IBALCC-RPDR 00016); adult female, Grande Sertão Veredas National Park [Parque Nacional Grande Setão Veredas], 15°11'12.1"S, 45°42'39.9"W, 6.ii.2018, D. Álvarez, manual capture (IBALCC-RPDR 00336).

***Tityusspelaeus* Moreno-González, Pinto-da-Rocha & Gallão, 2021.** Brazil, state of Goiás: adult female, Posse, Russão II cave, 1.iv.2007, R. Pinto-da-Rocha et al. (IBALCC-RPDR 00116).

***Tityusstigmurus* (Thorell, 1876)** Brazil, state of Pernambuco: adult female, Triunfo, 9.vii.2009, R. Pinto-da-Rocha, C. Bragagnolo & M. B. da Silva (IBALCC-RPDR 00170); adult female, Vitória de Santo Antão, 08°07'S, 35°23"W, 28.v.2008, H. Yamaguti, W. Porto & M. B. da Silva (IBALCC-RPDR 00218); adult male, Exu, 07°26'44"S, 39°44'21"W, 1.vi.2008, H. Yamaguti, W. Porto & M. B. da Silva (IBALCC-RPDR 00219);

***Tityussylviae* Lourenço, 2005**: Brazil, state of Amazonas: adult female (paratype), Jaú National Park [Parque Nacional do Jaú], Seringalzinho, pitfall, together with *Tityusdinizi* and *Tityussilvestris*, 01°52'34"S, 61°35'15"W, 1–8.viii.2001, I. Ghizoni Jr. (MNHN-RS-8620).

***Tityustrivittatus* Kraepelin, 1898**. Brazil, state of São Paulo: one adult male, Linhares, Fazenda Cupido, 2.x.1944, Schubart (MZSP 21775). State of Paraná: two females, Palmeira, xii.1852, Schubart (MZSP 21768).

## References

[B1] Ab’Saber NA (1977) Os domínios morfoclimáticos na América do Sul. Geomorfologia 52: 1–21.

[B2] Armas LFD Antún AJA (2004) Adiciones al género *Tityus* C. L. Koch, 1836 en República Dominicana, con la descripción de dos especies nuevas. Revista Ibérica de Aracnología 10: 53–64.

[B3] Astrin JJ Höfer H Spelda J Holstein J Bayer S Hendrich L Huber BA Kielhorn K-H Krammer H-J Lemke M (2016) Towards a DNA barcode reference database for spiders and harvestmen of Germany. PLoS ONE 11: e0162624. 10.1371/journal.pone.0162624 PMC5040438 27681175

[B4] Bichuette ME Trajano E (2006) Morphology and distribution of the cave knifefish *Eigenmanniavicentespelaea* Triques, 1996 (Gymnotiformes: Sternopygidae) from Central Brazil, with an expanded diagnosis and comments on subterranean evolution. Neotropical Ichthyology 4: 99–105. 10.1590/S1679-62252006000100011

[B5] Bichuette ME Nascimento AR Von Schimonsky DM Gallão JE Resende LPA Zepon T (2017) Terrestrial fauna of the largest granitic cave from Southern Hemisphere, southeastern Brazil: A neglected habitat. Neotropical Biology and Conservation. 12: 75–90. 10.4013/nbc.2017.122.01

[B6] Blomquist GJ Tittiger C Jurenka R (2020) Cuticular hydrocarbons and pheromones of arthropods. In: Hydrocarbons, oils and lipids: diversity, origin, chemistry, and fate. Handbook of Hydrocarbon and Lipid Microbiology. Springer Nature, Switzerland, 213–244. 10.1007/978-3-319-90569-3_11

[B7] Borges A Graham MR (2016) Phylogenetics of scorpions of medical importance. In: Gopalakrishnakone P Calvete JJ (Eds) , Venom Genomics and Proteomics.Toxinology. Springer Netherlands, Dordrecht, 81–104. 10.1007/978-94-007-6416-3_36

[B8] Borges A Jowers MJ Bónoli S De Sousa L (2012) Scorpions from the primeval subgenus Archaeotityus produce putative homologs of *Tityusserrulatus* toxins active on voltage-gated sodium channels. Journal of Venomous Animals and Toxins including Tropical Diseases 18: 432–440. 10.1590/S1678-91992012000400012

[B9] Borges A Bermingham E Herrera N Alfonzo MJ Sanjur OI (2010) Molecular systematics of the neotropical scorpion genus *Tityus* (Buthidae): The historical biogeography and venom antigenic diversity of toxic Venezuelan species. Toxicon 55: 436–454. 10.1016/j.toxicon.2009.09.011 19799925

[B10] Botero-Trujillo R Flórez E (2014) A new species of *Tityus* (Scorpiones, Buthidae) from El Edén Cave, Colombia. Zootaxa 3796: 108–120. 10.11646/zootaxa.3796.1.5 24870667

[B11] Cordeiro LM Borghezan R Trajano E (2014) Subterranean biodiversity in the serra da Bodoquena karst area, paraguay river basin, Mato Grosso do Sul, Southwestern Brazil. Biota Neotropica 14: 1–28. 10.1590/1676-06032014011414

[B12] Costa-Leonardo AM Casarin FE Lima JT (2009) Chemical communication in Isoptera. Neotropical Entomology 38: 1–6. 10.1590/S1519-566X2009000100001 19347093

[B13] Esposito LA Yamaguti HY Souza CA Pinto-Da-Rocha R Prendini L (2017) Systematic revision of the neotropical club-tailed scorpions, *Physoctonus*, *Rhopalurus*, and *Troglorhopalurus*, revalidation of *Heteroctenus*, and descriptions of two new genera and three New Species (Buthidae: Rhopalurusinae). Bulletin of the American Museum of Natural History 415: 1–136. 10.1206/0003-0090-415.1.1

[B14] Esposito LA Yamaguti HY Pinto-da-Rocha R Prendini L (2018) Plucking with the plectrum: phylogeny of the New World buthid scorpion subfamily Centruroidinae Kraus, 1955 (Scorpiones: Buthidae) reveals evolution of three pecten-sternite stridulation organs. Arthropod Systematics & Phylogeny 76: 87–122.

[B15] Fet V Lowe G (2000) Family Buthidae C. L. Koch, 1837. In: Catalog of the Scorpions of the World. The New York Entomological Society, New York, 54–286.

[B16] Fetzner Jr JW (1999) Extracting high-quality DNA from shed reptile skins: a simplified method. Biotechniques 26: 1052–1054. 10.2144/99266bm09 10376138

[B17] Francke OF (1977) Scorpions of the genus *Diplocentrus* from Oaxaca, Mexico (Scorpionida, Diplocentridae). Journal of Arachnology 4: 145–200.

[B18] Francke OF Stockwell SA (1987) Scorpions (Arachnida) from Costa Rica. Special Publications The Museum of Texas Tech University, Austin, 63 pp. 10.5962/bhl.title.156482

[B19] Gallão JE Bichuette ME (2016) On the enigmatic troglobitic scorpion *Troglorhopalurustranslucidus*: distribution, description of adult females, life history and comments on *Rhopaluruslacrau* (Scorpiones: Buthidae). Zoologia (Curitiba) 33: 1–13. 10.1590/s1984-4689zool-20150193

[B20] Gantenbein B Fet V Largiadèr CR Scholl A (1999) First DNA phylogeny of *Euscorpius* Thorell, 1876 (Scorpiones, Euscorpiidae) and its bearing on taxonomy and biogeography of this genus. Biogeographica (Paris) 75: 49–65.

[B21] Gnaspini P Trajano E (1994) Brazilian cave invertebrates, with a checklist of troglomorphic taxa. Revista Brasileira de Entomologia 38: 549–584.

[B22] González-Sponga MA (1974) Dos nuevas especies de alacranes del género *Tityus* en las cuevas venezolanas (Scorpionida: Buthidae). Boletín de la Sociedad Venezolana de Espeleología 5: 55–72.

[B23] Junior SDTP (1932) Considerações a respeito da systematica geral do genero *Tityus* e do *Tityusbahiensis* em particular. Revista de Agricultura 7: 295–306.

[B24] Katoh K Standley DM (2013) MAFFT multiple sequence alignment software version 7: improvements in performance and usability. Molecular Biology and Evolution 30: 772–780. 10.1093/molbev/mst010 23329690 PMC3603318

[B25] Kocher TD Thomas WK Meyer A Edwards SV Pääbo S Villablanca FX Wilson AC (1989) Dynamics of mitochondrial DNA evolution in animals: amplification and sequencing with conserved primers. Proceedings of the National Academy of Sciences 86: 6196–6200. 10.1073/pnas.86.16.6196 PMC297804 2762322

[B26] Kraepelin K (1895) Nachtrag zu Theil I der Revision der Scorpione. Jahrbuch der Hamburgischen Wissenschaftlichen Anstalten 12: 73–96.

[B27] Loria SF Prendini L (2014) Homology of the lateral eyes of scorpiones: A six-ocellus model. PLoS ONE 9: 1–30. 10.1371/journal.pone.0112913 PMC4254604 25470485

[B28] Lourenço WR (1981) Scorpions cavernicoles de l’Equateur: *Tityusdemangei* n. sp. et *Ananterisashmolei* n. sp. (Buthidae): *Troglotayosicusvachoni* n. gen., n. sp. (Chactidae), Scorpion troglobie. Bulletin du Museum national d’histoire naturelle. Section A: Zoologie, biologie et ecologie animales 2: 635–662.

[B29] Lourenço WR (1984) Analyse taxonomique des scorpions du groupe *Tityusclathratus* Koch, 1845 (Scorpiones, Buthidae). Bulletin du Muséum national d’histoire naturelle. Section A, Zoologie, biologie et écologie animales 6: 349–360.

[B30] Lourenço WR (1999) Origines et affinities des scorpions des Grandes Antilles: le cas particular des elements de la famille des Buthidae. Biogeographica 75: 131–144.

[B31] Lourenço WR (2000) Synopsis of the colombian species of *Tityus* Koch (Chelicerata, Scorpiones, Buthidae), with descriptions of three new species. Journal of Natural History 34: 449–461. 10.1080/002229300299561

[B32] Lourenço WR (2002a) 4.9 Scorpiones. In: Amazonian Arachnida and Myriapoda. Faunistica N° 24. Pensoft Publishers, Sofia-Moscow, 399–438.

[B33] Lourenço WR (2002b) Scorpions of Brazil. Les editions de l’If Paris, 304 pp.

[B34] Lourenço WR (2006) Une nouvelle proposition de découpage sous-générique du genre “*Tityus*” C.L. Koch, 1836 (Scorpiones, Buthidae). Boletín de la Sociedad Entomológica Aragonesa 39: 55–67.

[B35] Lourenço WR (2011) The distribution of noxious species of scorpions in Brazilian Amazonia: the genus Tityus CL Koch, 1836, subgenus Atreus Gervais, 1843 (Scorpiones, Buthidae). Entomologische Mitteilungen aus dem Zoologischen Museum Hamburg 15: 287–301.

[B36] Lourenço WR (2015) What do we know about some of the most conspicuous scorpion species of the genus *Tityus*? A historical approach. Journal of Venomous Animals and Toxins Including Tropical Diseases 21: 1–12. 10.1186/s40409-015-0016-9 PMC4470017 26085830

[B37] Lourenço WR (2016) Une nouvelle espèce de *Tityus* CL Koch, 1836 (Scorpiones: Buthidae), collectée par Jean A. Vellard dans l’ancien Etat de Goiás, aujourd’hui Tocantins, Brésil. Revista Ibérica de Aracnología: 75–78.

[B38] Lourenço WR (2019) New insights on the scorpion species of the “*Tityustrivittatus* group” of subgenus Tityus CL Koch, 1836 (Scorpiones: Buthidae). Revista Ibérica de Aracnología: 119–125.

[B39] Lourenço WR Eickstedt VRD von (1984) Descricão de uma espécie nova de *Tityus* coletada no Estado da Bahia, Brasil (Scorpiones, Buthidae). Journal of Arachnology 12: 55–60.

[B40] Lourenço WR Francke OF (1985) Révision des connaissances sur les scorpions cavernicoles (troglobies) (Arachnida, Scorpions). Mèmoires Biospéologiques 12: 3–7.

[B41] Lourenco WR Pinto-da-Rocha R (1997) A reappraisal of the geographic distribution of the genus *Rhopalurus* Thorell (Scorpiones, Buthidae) and description of two new species. Biogeographica 73: 181–191.

[B42] Lourenço WR Duhem B (2010) Buthid scorpions found in caves; a new species of *Isometrus* Ehrenberg, 1828 (Scorpiones, Buthidae) from southern Vietnam. Comptes Rendus – Biologies 333: 631–636. 10.1016/j.crvi.2010.05.005 20688284

[B43] Lourenço WR Pham DS (2013) First record of a cave species of *Euscorpiops* Vachon from Viet Nam (Scorpiones, Euscorpiidae, Scorpiopinae). Comptes Rendus – Biologies 336: 370–374. 10.1016/j.crvi.2013.06.005 23932257

[B44] Lourenço WR Knox MB Magalhães ED (1997) Redescription of *Tityusblaseri* (Scorpiones: Buthidae) from Goiás, Brazil. Revista de Biología Tropical 45: 1579–1582.

[B45] Lourenço WR Cabral BC Ramos EB (2004) Confirmation of *Tiyusconfluens* Borelli, 1899 (Scorpiones, Buthidae) in Brazil and description of a new subspecies from the State of Mato Grosso do Sul. Boletín de la Sociedad Entomológica Aragonesa 34: 27–30.

[B46] Mcwest KJ (2009) Tarsal spinules and setae of vaejovid scorpions (Scorpiones: Vaejovidae). Zootaxa 2001: 1–126.

[B47] Mello-Leitão C de (1945) Escorpiões sul-americanos. Arquivos do Museu Nacional 40: 7–468.

[B48] Minh BQ Schmidt HA Chernomor O Schrempf D Woodhams MD Von Haeseler A Lanfear R (2020) IQ-TREE 2: New models and efficient methods for phylogenetic inference in the genomic era. Molecular Biology and Evolution 37: 1530–1534. 10.1093/molbev/msaa015 32011700 PMC7182206

[B49] Moreno-González JA González OR Flórez DE (2019) Taxonomic revision of the Colombian Tityus (Archaeotityus) (Scorpiones, Buthidae) species: A morphological and morphometric approach, with a description of a new species. Zootaxa 4660: 1–94. 10.11646/zootaxa.4660.1.1 31716727

[B50] Moreno-González JA (2021) Phylogenetic analysis of the *Tityusclathratus* species-group and other species-groups and subgenera of *Tityus* (Scorpiones: Buthidae) based on molecular and morphological characters [In portuguese: *A*nálise filogenética do grupo de espécies *Tityusclathratus* e outros grupos e subgêneros de *Tityus* (Scorpiones: Buthidae) baseada em caracteres moleculares e morfológicos]. Ph.D. dissertation. Universidade de São Paulo, São Paulo, 250 pp.

[B51] Nimer E (1979) Climatologia do Brasil. Vol. 4. Rio de Janeiro: SUPREN.

[B52] Ojanguren-Affilastro AA Adilardi RS Mattoni CI Ramírez MJ Ceccarelli FS (2017a) Dated phylogenetic studies of the southernmost American buthids (Scorpiones; Buthidae). Molecular Phylogenetics and Evolution 110: 39–49. 10.1016/j.ympev.2017.02.018 28259729

[B53] Ojanguren-Affilastro AA Adilardi RS Cajade R Ramírez MJ Ceccarelli FS Mola LM (2017b) Multiple approaches to understanding the taxonomic status of an enigmatic new scorpion species of the genus *Tityus* (Buthidae) from the biogeographic island of Paraje Tres Cerros (Argentina). PLoS ONE 12: 1–24. 10.1371/journal.pone.0181337 PMC5529008 28746406

[B54] Ojanguren-Affilastro AA Kochalka J Guerrero-Orellana G Garcete-Barrett B Roodt AR Borges A Ceccarelli S (2021) Redefinition of the identity and phylogenetic position of *Tityustrivittatus* Kraepelin 1898, and description of *Tityuscarrilloi* n. sp. (Scorpiones; Buthidae), the most medically important scorpion of southern South America. Revista del Museo Argentino de Ciencias Naturales, nueva serie 23: 27–55. 10.22179/REVMACN.23.714

[B55] Outeda-Jorge S Mello T Pinto-da-Rocha R (2009) Litter size, effects of maternal body size, and date of birth in South American scorpions (Arachnida: Scorpiones). Zoologia (Curitiba) 26: 43–53. 10.1590/s1984-46702009000100008

[B56] Pelosi P Iovinella I Felicioli A Dani FR (2014) Soluble proteins of chemical communication: an overview across arthropods. Frontiers in Physiology 5: e320. 10.3389/fphys.2014.00320 PMC4145409 25221516

[B57] Pinto-da-Rocha R (1995) Sinopse da fauna cavernícola do Brasil (1907–1994). Papéis Avulsos de Zoologia 39: 61–173.

[B58] Pinto-da-Rocha R Bragagnolo C Marques FP Antunes Junior M (2014) Phylogeny of harvestmen family Gonyleptidae inferred from a multilocus approach (Arachnida: Opiliones). Cladistics 30: 519–539. 10.1111/cla.12065 34772271

[B59] Prendini L (2000) Phylogeny and classification of the superfamily Scorpionoidea Latreille 1802 (Chelicerata, Scorpiones): An exemplar approach. Cladistics 16: 1–78. 10.1006/clad.1999.0127 34902920

[B60] Prendini L (2001) Further additions to the scorpion fauna of Trinidad and Tobago. Journal of Arachnology 29: 173–188. 10.1636/0161-8202(2001)029[0173:FATTSF]2.0.CO;2

[B61] Prendini L (2003a) Discovery of the male of *Parabuthusmuelleri*, and implications for the phylogeny of *Parabuthus* (Scorpiones: Buthidae). American Museum Novitates 3408: 1–24. 10.1206/0003-0082(2003)408<0001:DOTMOP>2.0.CO;2

[B62] Prendini L (2003b) Revision of the genus *Lisposoma* Lawrence, 1928 (Scorpiones: Bothriuridae). Insect Systematics & Evolution 34: 241–264. 10.1163/187631203788964764

[B63] Prendini L Francke OF Vignoli V (2010) Troglomorphism, trichobothriotaxy and typhlochactid phylogeny (Scorpiones, Chactoidea): more evidence that troglobitism is not an evolutionary dead-end. Cladistics 26: 117–142. 10.1111/j.1096-0031.2009.00277.x 34875760

[B64] Prendini L Ehrenthal VL Loria SF (2021) Systematics of the relictual Asian scorpion family Pseudochactidae Gromov, 1998, with a review of cavernicolous, troglobitic, and troglomorphic scorpions. Bulletin of the American Museum of Natural History 453: 1–149. 10.1206/0003-0090.453.1.1

[B65] Racovitza EG (1907) Essai sur les problemes biospeologiques. Archives des Maladies du Coeur et des Vaisseaux 4e serie 6: 371–48.

[B66] Reddell JR (2012) Spiders and related groups. In: Ecosystems of the World, Subterranean Ecosystems. Elsevier Academic Press, Amsterdam, 554–564. 10.1016/b978-0-12-814124-3.00118-7.

[B67] Rein JO (2021) The Scorpion Files. Trondheim: Norwegian University of Science and Technology. https://www.ntnu.no/ub/scorpion-files/

[B68] Richard F-J Hunt JH (2013) Intracolony chemical communication in social insects. Insectes Sociaux 60: 275–291. 10.1007/s00040-013-0306-6

[B69] Román JP García F Medina D Vásquez M García J Graham MR Romero-Alvarez D Pardal PP de O Ishikawa EAY Borges A (2018) Scorpion envenoming in Morona Santiago, Amazonian Ecuador: Molecular phylogenetics confirms involvement of the *Tityusobscurus* group. Acta Tropica 178: 1–9. 10.1016/j.actatropica.2017.10.014 29079184

[B70] Schiestl FP (2010) The evolution of floral scent and insect chemical communication. Ecology Letters 13: 643–656. 10.1111/j.1461-0248.2010.01451.x 20337694

[B71] Schiner JR (1854) Fauna der Adelsberger-, Luegger-, und Magdalenen- Grotte. In: Die Grotten und Höhlen von Adelsberg, Lueg, Planina und Laas. Braunmüller, Wien, 231–272.

[B72] Schulmeister S (2003) Simultaneous analysis of basal Hymenoptera (Insecta): introducing robust-choice sensitivity analysis. Biological Journal of the Linnean Society 79: 245–275. 10.1046/j.1095-8312.2003.00233.x

[B73] Schwendinger PJ Giribet G (2005) The systematics of the south-east Asian genus *Fangensis* Rambla (Opiliones: Cyphophthalmi: Stylocellidae). Invertebrate Systematics 19: 297–323. 10.1071/IS05023

[B74] Simon C Frati F Beckenbach A Crespi B Liu H Flook P (1994) Evolution, weighting, and phylogenetic utility of mitochondrial gene sequences and a compilation of conserved polymerase chain reaction primers. Annals of the Entomological Society of America 87: 651–701. 10.1093/aesa/87.6.651

[B75] Sissom WD Reddell JR (2009) Cave scorpions of Mexico and the United States. Texas Memorial Museum Speleological Monographs 7: 19–32.

[B76] Sissom WD Polis GA Watt DD (1990) Field and laboratory methods. In: The Biology of Scorpions. Standford University Press, California, 445–461.

[B77] Soleglad ME Fet V (2003) High-level systematics and phylogeny of the extant scorpions (Scorpiones: Orthosterni). Euscorpius 11: 1–56. 10.18590/euscorpius.2003.vol2003.iss11.1

[B78] Souza CARDE Candido DM Lucas SM Brescovit AD (2009) On the *Tityusstigmurus* complex (Scorpiones, Buthidae). Zootaxa 38: 1–38.

[B79] Stahnke HL (1970) Scorpion nomenclature and mensuration. Entomological News 81: 297–316. 5417256

[B80] Tencatt LFC Bichuette ME (2017) *Aspidorasmephisto*, new species: the first troglobitic Callichthyidae (Teleostei: Siluriformes) from South America. PLoS ONE 12: e0171309. 10.1371/journal.pone.0171309 PMC5331963 28248959

[B81] Teruel R García LF (2008a) Rare or poorly known scorpions from Colombia. I. Redescription of *Tityusmacrochirus* Pocock, 1897 (Scorpiones: Buthidae). Euscorpius 63: 1–11. 10.18590/euscorpius.2008.vol2008.iss63.1

[B82] Teruel R García LF (2008b) Rare or poorly known scorpions from Colombia. II. Redescription of *Tityuscolumbianus* (Thorell , 1876) (Scorpiones: Buthidae). Euscorpius 64: 1–14. 10.18590/euscorpius.2008.vol2008.iss64.1

[B83] Trajano E (1987) Fauna cavernícola brasileira: composição e caracterização preliminar. Revista Brasileira de Zoologia 3: 533–561. 10.1590/S0101-81751986000400004

[B84] Trajano E (2012) Ecological classification of subterranean organisms. In: White WB Culver DC (Eds) Encyclopedia of Caves (Second Edition).Academic Press, Amsterdam, 275–277. 10.1016/B978-0-12-383832-2.00035-9

[B85] Trajano E Moreira JRA (1991) Estudo da fauna de cavernas da Província Espeleológica Arenítica Altamira-ltaituba, Pará. Revista Brasileira de Zoologia 51: 13–29.

[B86] Trajano E Carvalho MR (2017) Towards a biologically meaningful classification of subterranean organisms: a critical analysis of the Schiner-Racovitza system from a historical perspective, difficulties of its application and implications for conservation. Subterranean Biology 22: 1–26. 10.3897/subtbiol.22.9759.figure1

[B87] Vachon M (1963) De l’utilité, en systématique, d’une nomenclature des dents des chélicères chez les Scorpions. Bulletin du Muséum national d’Histoire naturelle, Paris: 161–166.

[B88] Vachon M (1974) Etude des caractères utilisés pour classer les familles et les genres de Scorpions (Arachnides). 1. La trichobothriotaxie en arachnologie. Sigles trichobothriaux et types de trichobothriotaxie chez les Scorpions. Bulletin du Muséum national d’Histoire naturelle, Paris 140: 857–958.

[B89] Vachon M (1975) Sur l’utilisation de la trichobothriotaxie du bras des pédipalpes des Scorpions (Arachnides) dans le classe- ment des genres de la famille des Buthidae Simon. Comptes Rendus des séances de l’Académie des Sciences, Paris, série D 281: 1597–1599.

[B90] Vaidya G Lohman DJ Meier R (2011) SequenceMatrix: concatenation software for the fast assembly of multi-gene datasets with character set and codon information. Cladistics 27: 171–180. 10.1111/j.1096-0031.2010.00329.x 34875773

[B91] Volschenk ES Prendini L (2008) *Aopsoncodactylus*, gen. et sp. nov., the first troglobitic urodacid (Urodacidae: Scorpiones), with a re-assessment of cavernicolous, troglobitic and troglomorphic scorpions. Invertebrate Systematics 22: 235–257. 10.1071/IS06054

[B92] Whiting MF Carpenter JC Wheeler QD Wheeler WC (1997) The Strepsiptera problem: phylogeny of the holometabolous insect orders inferred from 18S and 28S ribosomal DNA sequences and morphology. Systematic Biology 46: 1–68. 10.1093/sysbio/46.1.1 11975347

